# Exploring the anti-microbial potential of benzylated imidazolium salts: synthesis, docking studies, and biological evaluation

**DOI:** 10.1039/d5ra04144a

**Published:** 2025-09-24

**Authors:** Pandurangan Ganapathi, Kilivelu Ganesan, Nallusamy Vijaykanth, Srisailas Muthialu, Shiek S. S. J. Ahmed, Mohammed Mujahid Alam, Mohamed Hussien

**Affiliations:** a PG & Research Department of Chemistry, Presidency College (Autonomous) Chennai 600005 India kiliveluganesan@yahoo.co.in; b Filaria/Malaria Clinic, Government Hospital Valangaiman Thiruvarur District-612804 Tamil Nadu India; c Vasi Pharma LLC, 150 N Research Campus Drive Kannapolis NC 28081 USA; d Drug Discovery and Multi-omics Laboratory, Faculty of Allied Health Sciences, Chettinad Hospital and Research Institute, Chettinad Academy of Research and Education Kelambakkam-603103 Tamil Nadu India; e Department of Chemistry, College of Science, King Khalid University P.O. Box 9004 Abha 61413 Saudi Arabia

## Abstract

Dibenzyl methyl-substituted imidazolium salts were synthesized from readily available starting materials using conventional methods. Initially, benzyl bromide derivatives were employed to alkylate 2-methyl-5-nitroimidazole under reflux conditions, yielding mono-, di-, and tri-meric imidazolium salts in high yields. Subsequent anion–exchange reactions produced compounds with yields of 83–89%, while solid-phase, silica-supported processes further enhanced yields to 89–96%. The antibacterial activity of these compounds against six human pathogens (*Escherichia coli*, *Klebsiella pneumoniae*, *Pseudomonas aeruginosa*, *Proteus vulgaris*, *Staphylococcus aureus*, and *Enterococcus faecalis*) was evaluated using well diffusion and broth microdilution techniques. Notably, nitro-substituted imidazolium salts demonstrated significant antibacterial activity, with bromide variants exhibiting the strongest inhibition. Minimum inhibitory concentration values revealed potent bactericidal effects. In terms of ADMET properties, all synthesized compounds exhibited favorable profiles, including good gastrointestinal absorption, skin permeation, and minimal inhibitory effects on key cytochrome P450 enzymes. Molecular docking analysis revealed significant binding affinities, particularly for compounds 4 and 6, with bacterial proteins, highlighting key interactions such as hydrogen bonding and π-alkyl stacking. Molecular dynamic simulations of top compounds against bacterial target proteins exhibited stable interactions and conformations throughout 100 ns trajectories. These synthesized compounds showed promising antibacterial properties, warranting further investigation for potential therapeutic applications.

## Abstract 2

Dibenzyl methyl-substituted imidazolium salts were synthesized from readily available starting materials using conventional and solvent free solid supported methods.

## Graphical abstract

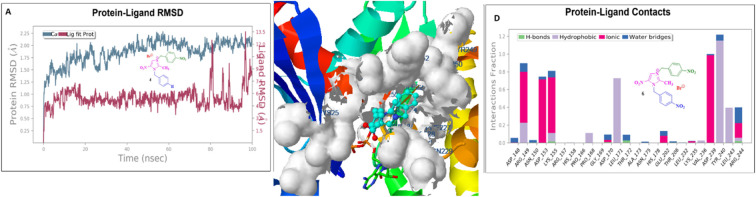

## Introduction

Ionic liquids are salts composed of simple counter anions such as bromide, chloride, sulphate, acetate, tetrafluoroborate, and hexafluoro phosphate as well as complex (or) organic cations such as the quaternary ammonium/pyrrolidinium/pyridinium/imidazolium cation.^1^ Self-assembling behaviors of phenyl-alkene-substituted, medium-alkyl-chain-substituted imidazolium salts were better than those of the shorter-alkyl-chain-linked imidazolium salt. Ionic liquids have been used as CO_2_ absorbents,^2–4^ lubricants,^5,6^ solvents,^7,8^ catalysts,^9^ electrolytes,^10^ surfactants,^18,19^ and antiseptic agents^12^ as well as for biotechnological^11^ and pharmaceutical applications^13–17^ due to their exceptional behaviors consisting of the combination of an organic cation with an inorganic anion, an inorganic cation with an organic anion, or an organic cation with an organic anion.^20–23^ Antibacterial and antifungal activities of 1-alkyl-3-methyl imidazolium salts and ternary lanthanum coordination polymer were assessed against Gram-positive and Gram-negative bacteria.^24–27^ For ascomycetes and basidiomycetes fungi, the larger alkyl chain linked to imidazolium chloride showed better antibacterial and antifungal responses than shorter alkyl substituents.^28^ Optically active amino acids substituted for amide-based imidazolium and pyridinium salts are synthesized using a green synthetic approach. The aggregation, biodegradation, bacterial and fungal toxicities of these compounds were studied.^29^ An organic hydrophobic cation (quaternary ammonium ion) with a hydrophilic organic anion (dicamba) exhibited volatility, thermal stability and strong herbicidal properties.^30^ Marta *et al.* reported that various shorter/longer alkyl-substituted imidazolium cations with various simple and complex anions of surface-active ionic liquids are used as crop production agents.^31^ Nowadays, the application of ionic liquids in medicinal field is enormous due to their unique properties, such as high purity, easy storage, greater thermal stability and distinct solubility behaviours. Imidazolium/pyridinium-based ionic liquids have played a significant role in the development of biomaterials/modifications (or) alteration of drug delivery properties.^32^ Joanna and coworkers prepared some novel non-steroidal anti-inflammatory drug molecules from l-amino acid alkyl-substituted ammonium cations, which show effective responses in anti-inflammatory, photo-protective and anti-ageing effects.^33^ A simple/alkyl substituted-4-benyl morpholinium cation with various organic anions showed phytotoxicity and wetting weed surface activities.^34^ Based on the available reports, the aim is to design and synthesize a series of compounds using both conventional and solvent-free methods, employing a muffle furnace. The synthesized compounds will be evaluated for their anti-bacterial activity, and further investigated through molecular docking and simulation studies.

## Results and discussion

**Scheme 1:**
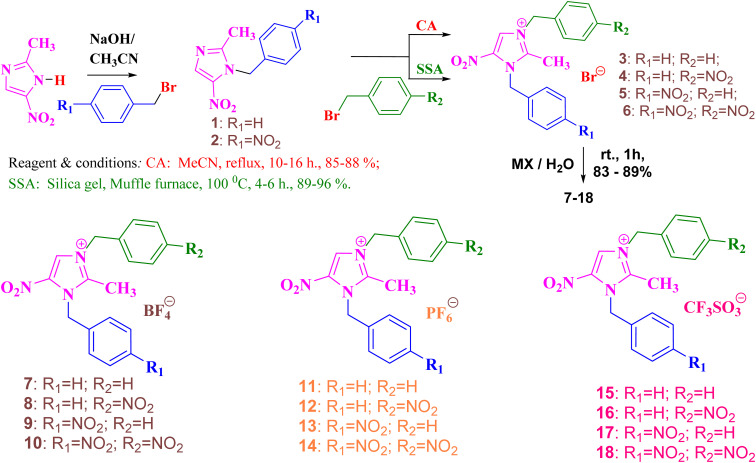
Synthesis of flexible substituted monomeric imidazolium salts under multiple routes.

2-Methyl-5-nitroimidazole is treated with 1.05 equivalent of benzyl bromide/4-nitro benzyl bromide in the presence of NaOH/CH_3_CN under refluxing to afford the compounds 1/2 in 95–97% yield. *N*-Alkylation of compound 1/2 (1.0 equiv.) is reacted with benzyl bromide/4-nitrobenzylbromide (1.05 equiv.) in the presence of dry CH_3_CN under refluxing condition for about 10–16 h to give imidazolium bromide 3–6 in 85–88% yield. The same reaction is then tried under the solvent-free silica supported approach. The uniformly mixed *N*-alkylation products of compounds 1/2 (1.0 equiv.) and benzyl bromide/4-nitrobenzylbromide (1.05 equiv.) are kept in a muffle furnace with silica gel (80–120 mesh) at 100 °C for 4–6 h to give compounds 3–6 in 89–96% yield (Scheme 1). DBMNIB 3–6 is subjected to the anion exchange reaction with various inorganic salts in the presence of deionized water for 1 h to give the anion exchanged products of compounds 7–18 in 83–89% yield. After the anion-exchanged reaction, we used the Soxhlet extraction to remove metal bromide from imidazolium salts using 100 mL of dry THF for about 1 h reflux to give the respective imidazolium salts in quantitative yield (Scheme 1).

### Bacterial cultures

Six pathogenic bacteria are used in this study as test microorganisms, namely, *Escherichia coli, Klebsiella pneumoniae, Pseudomonas aeruginosa, Proteus vulgaris, Staphylococcus aureus* and *Enterococcus faecalis*. The bacterial cultures were obtained from the Department of Microbiology, Presidency College, Chennai, India. The bacteria are subcultured using Nutrient agar (Himedia, India) and stored at 4 °C until required for the study.

### Sterility test

A loopful of imidazolium salts were inoculated into Nutrient agar and Sabouraud Dextrose agar plates, and incubated at 37 °C and 20 °C for 24 h and 72 h, respectively. All compounds passed the sterility control.

### Antibacterial studies

In the literature, 1-alkyl quinolinium types of ionic liquids showed antibacterial activities against Gram-positive/negative pathogens.^35^ Docherty *et al.* have mentioned that the bacterial screenings of their synthesized quinolinium salts are purely based on the length of the side chain.^36–40^ While moving from carbon number 8 to 16, the antibacterial responses will be enhanced. Based on this report, the antibacterial activity was screened against both Gram-positive and Gram-negative pathogens. The mono- and dimeric imidazolium salts exhibited excellent inhibition compared to available reports.^35^ The antibacterial activities of imidazolium salts were studied by well/disc diffusion methods using Mueller Hinton Agar (MHA).^41^ The stock solutions of the mono and dimeric imidazolium salts were prepared as 1 mg mL^−1^, and dilutions of 25 μg per well, 50 μg per well, 75 μg per well and 100 μg per well concentrations were prepared using dimethyl sulfoxide (DMSO) as the solvent (Table 1). The bacterial inoculum was adjusted to a scale factor of 0.5 of the McFarland standard.^42^ The dilutions of the imidazolium salts were loaded into the respective wells of the MHA plate. Gentamycin (30 μg per well), nalidixic acid (30 μg per well), oflaxacin (30 μg per well), ciproflaxacin (30 μg per well) and amikacin (30 μg per well) were used as standard drugs for comparison. The MHA plates were incubated at 37 °C for 18–24 h. The zone of inhibition was measured in mm using a Vernier caliper and compared with the standard drug disc. Izabelle and co-workers reported that dicationic imidazolium-based ionic liquids exhibited IC_50_ values (dose to inhibit 50% of enzymatic activity) greater than those of the reported monocationic ionic liquids.^43^ Thus, the imidazolium salts in question appear to be more effective than those described in the literature.^44–51^

**Table 1:** Antibacterial screening of monomeric imidazolium salts 3–18 with different counter anions against human pathogens under the disc diffusion method

S. No.	Standard drug (30 μg per well)/imidazolium cation with different anions (μg per well)		Zone of inhibition (mm)					
			Gram-negative organism				Gram-positive organism	
			*E. coli*	*K. pneumoniae*	*P. aeruginosa*	*P. vulgaris*	*S. aureus*	*E. faecalis*
1	Gentamycin		20	14	20	18	20	16
2	Amikacin		15	20	22	20	20	18
3	Nalidicxic acid		7	—	15	—	15	—
4	Oflaxacin		20	17	28	8	22	22
5	Ciproflaxacin		23	23	30	—	25	25
6	3	25	5.5	5	5	—	7	—
		50	6	5.5	6.5	5	8	—
		75	6.5	6	7.5	6	10	5
		100	7.5	7	9	6.5	12	6
7	7	25	5	5	9	—	6.5	—
		50	5.5	5.5	10.5	5	7.5	—
		75	6.5	6	12	5.5	9	—
		100	7.5	7	14	6.5	10.5	5.5
8	8	25	5	6	7.5	—	6.5	—
		50	5.5	7	9	5	8	—
		75	6	8.5	10	6.5	9.5	—
		100	6.5	10	11	8	11	5
9	9	25	5	6	7	—	6	—
		50	5	7.5	8	5	7	—
		75	5.5	9	9.5	6	8.5	—
		100	6	10	10.5	7.5	10	5.5
10	4	25	7	6.5	9.5	5	7.5	—
		50	8	7	11	5.5	8.5	—
		75	9.5	8	12	6.5	10	5
		100	10.5	10	13.5	8	12	6
11	10	25	5.5	5.5	8.5	—	7	—
		50	6	6.5	10	5.5	8.5	—
		75	7	8	11	7	9.5	—
		100	8.5	10	12.5	8.5	11	5.5
12	11	25	5	5.5	8	—	6.5	—
		50	6.5	7	9	5	8	—
		75	7.5	8.5	10.5	6	9.5	—
		100	9	10.5	12	7.5	10.5	5
13	12	25	5.5	5	8.5	—	6.5	—
		50	6.5	6	10	5.5	8	—
		75	7	7	12.5	7	9.5	—
		100	8.5	9.5	14	8	11	5

### Determination of the minimum inhibitory concentration

Minimum inhibitory concentration (MIC) and minimum bacterial concentration (MBC) were determined by micro dilution method using Mueller Hinton Broth (MHB).^52^ A stock solution (1 mg mL^−1^) of the imidazolium salts and dilutions containing 10 μg per well, 20 μg per well, 30 μg per well, 40 μg per well, 50 μg per well, 60 μg per well, 70 μg per well and 80 μg per well concentrations were prepared using MHB. 100 μl of each dilution was loaded in the respective well of the micro titre plate. 100 μl of MHB was used as a control. Gentamycin, amikacin, nalidicxic acid and oflaxacin, ciproflaxacin were used as standard drugs.^53^ The plate was incubated at 37 °C for 18–24 h. The dilution showed that no bacterial growth was formed, demonstrating bacterial growth inhibition and inhibitory activities of the imidazolium salts.

### Recovery plate technique

20 μl of each well was streaked onto the sterile nutrient agar plates. The plates were incubated at 37 °C for 18–24 h. The presence of growth was observed for imidazolium salts which have no bactericidal activities, and the absence of growth was observed for imidazolium salts showing bactericidal activities.

### Microdilution technique

The MIC and MBC of the synthesized imidazolium salts against Gram-negative/Gram-positive human pathogens were studied under the microdilution technique.^54,55^ We have observed that BNBMNIB 6a shows excellent antimicrobial activity against *E. coli* and *P. vulgaris* compared to that against *S. aureus*; other pathogens showed good to moderate activity (Table 2). The MIC and MBC values were determined for dimeric imidazolium salts 3–18 [10 μg per well], 20 μg per well, 30 μg per well, 40 μg per well, 50 μg per well, 60 μg per well, 70 μg per well, and 80 μg per well gentamycin, nalidixic acid, oflaxacin, ciproflaxacin, and amikacin] concentrations against Gram-negative *E. coli*, *K. pneumoniae*, *P. aeruginosa*, and *P. vulgaris* and Gram-positive *S. aureus*, and *E. faecalis* human pathogens.

**Table 2:** Antibacterial screening of monomeric imidazolium salts 14–18 with different counter anions against human pathogens under the disc diffusion method

S. No.	Standard drug (30 μg/well)/imidazolium cation with different anions (μg/well)		Zone of inhibition (mm)					
			Gram-negative organism				Gram-positive organism	
			*E. coli*	*K. pneumoniae*	*P. aeruginosa*	*P. vulgaris*	*S. aureus*	*E. faecalis*
1	Gentamycin		20	14	20	18	20	16
2	Amikacin		15	20	22	20	20	18
3	Nalidicxic acid		7	—	15	—	15	—
4	Oflaxacin		20	17	28	8	22	22
5	Ciproflaxacin		23	23	30	—	25	25
14	5	25	6.5	6.5	8	5.5	8.5	—
		50	7.5	8	9	7	10	—
		75	9	9	10.5	8	12	6
		100	10.5	11	12	9.5	13.5	7.5
15	13	25	6.5	6	7.5	5	10.5	—
		50	7.5	7	9	6	13	—
		75	8.5	8.5	10	7.5	15	—
		100	10	10.5	11.5	8.5	17	6
16	14	25	6	5.5	6	5	8.5	—
		50	7	7	7	6.5	9.5	—
		75	8.5	8	8.5	8	11	—
		100	10	9.5	10	9.5	12.5	6.5
17	15	25	5.5	6	7	5	7.5	—
		50	7	7	8	6	9	—
		75	8	8.5	9.5	7	10.5	—
		100	9.5	10	11	8.5	12	6
18	6	25	6.5	8.5	9	6.5	7.5	—
		50	8	10	10	8	9	—
		75	9	11.5	11.5	9.5	10.5	6.5
		100	11	13	13	11	13	7.5
19	16	25	5.5	7.5	8.5	6	7.5	—
		50	6	9	9.5	7	9	—
		75	7.5	10	11	8	10	—
		100	8.5	11.5	12.5	9.5	11.5	5
20	17	25	6	7	6.5	6.5	8	—
		50	7.5	8	8	8	9.5	—
		75	8.5	9	9.5	9.5	11	—
		100	10	10.5	11	11	12.5	5.5
21	18	25	6.5	10	9.5	6.5	8	—
		50	8	11.5	10.5	8	9	—
		75	9	13.5	12	9	10.5	—
		100	10.5	15.5	13	10.5	12	6

### Well diffusion technique

Antimicrobial activities of imidazolium salts against Gram-negative (*Escherichia coli*, *Klebsiella pneumoniae*, *Pseudomonas aeruginosa*, *Proteus vulgaris*) and Gram-positive (*Staphylococcus aureus*, *Enterococcus faecalis*) microorganisms were studied under the well diffusion technique.^56^ Antimicrobial screening was conducted by the well diffusion technique as the zone of inhibition with diameters. Imidazolium salts 3–18 were screened for their microbial activities under well diffusion method. Nearly sixteen imidazolium salts were prepared and exhibited excellent inhibition. Accordingly, these sixteen imidazolium salts were spread on a Petri dish plate and screened *via* well diffusion with the six test pathogens (Tables 1–3).

**Table 3:** Minimum inhibitory concentration of DBMNIB 3–18 by colorimetric method (μg per well)^a^

S. No.	Standard drug/compound	Monomeric imidazolium cations with different anions											
		Gram-negative organism								Gram-positive organism			
		*E. coli*		*K. pneumoniae*		*P. aeruginosa*		*P. vulgaris*		*S. aureus*		*E. faecalis*	
		MIC	MBC	MIC	MBC	MIC	MBC	MIC	MBC	MIC	MBC	MIC	MBC
1	Gentamicin	0.235	0.235	0.265	0.265	0.235	0.235	0.235	0.235	0.235	0.235	0.265	0.265
2	Nalidixic acid	0.235	0.235	0.235	0.235	0.235	0.235	0.235	0.235	0.235	0.235	0.235	0.235
3	Oflaxacin	0.235	0.235	0.265	0.265	0.235	0.235	0.275	0.275	0.235	0.235	0.235	0.235
4	Ciproflaxacin	0.235	0.235	0.235	0.235	0.235	0.235	0.295	0.295	0.235	0.235	0.235	0.235
5	Amikacin	0.235	0.235	0.235	0.235	0.235	0.235	0.235	0.235	0.235	0.235	0.235	0.235
6	DBMNIB 3	0.265	0.265	0.275	0.275	0.265	0.265	0.285	0.285	0.265	0.265	0.295	0.295
7	DBMNIT 7	0.265	0.265	0.285	0.285	0.265	0.265	0.285	0.285	0.265	0.265	0.295	0.295
8	3-NBBMNIT 8	0.265	0.265	0.285	0.285	0.265	0.265	0.285	0.285	0.265	0.265	0.295	0.295
9	1-NBBMNIT 9	0.275	0.275	0.285	0.285	0.265	0.265	0.285	0.285	0.265	0.265	0.295	0.295
10	3-NBBMNIB 4	0.265	0.265	0.275	0.275	0.265	0.265	0.285	0.285	0.265	0.265	0.295	0.295
11	BNBMNIT 10	0.265	0.265	0.285	0.285	0.265	0.265	0.285	0.285	0.265	0.265	0.295	0.295
12	DBMNIH 11	0.265	0.265	0.285	0.285	0.265	0.265	0.285	0.285	0.265	0.265	0.295	0.295
13	3-NBBMNIH 12	0.275	0.275	0.285	0.285	0.265	0.265	0.285	0.285	0.265	0.265	0.295	0.295
14	1-NBBMNIB 5	0.265	0.265	0.275	0.275	0.265	0.265	0.275	0.275	0.265	0.265	0.295	0.295
15	1-NBBMNIH13	0.265	0.265	0.275	0.275	0.265	0.265	0.275	0.275	0.265	0.265	0.295	0.295
16	BNBMNIH 14	0.275	0.275	0.275	0.275	0.265	0.265	0.275	0.275	0.265	0.265	0.295	0.295
17	DBMNIT 15	0.265	0.265	0.275	0.275	0.265	0.265	0.275	0.275	0.265	0.265	0.295	0.295
18	BNBMNIB 6	0.265	0.265	0.265	0.265	0.265	0.265	0.265	0.265	0.265	0.265	0.295	0.295
19	3-NBBMNIT 16	0.265	0.265	0.275	0.275	0.265	0.265	0.265	0.265	0.265	0.265	0.295	0.295
20	1-NBBMNIH 17	0.265	0.265	0.275	0.275	0.265	0.265	0.265	0.265	0.265	0.265	0.295	0.295
21	BNBMNIT 18	0.275	0.275	0.275	0.275	0.265	0.265	0.275	0.275	0.265	0.265	0.295	0.295

a Medium control = 0.07 (medium only), organism control = 0.31 (medium + organism), drug control = 0.22 (medium + drug).

**Fig. 1:**
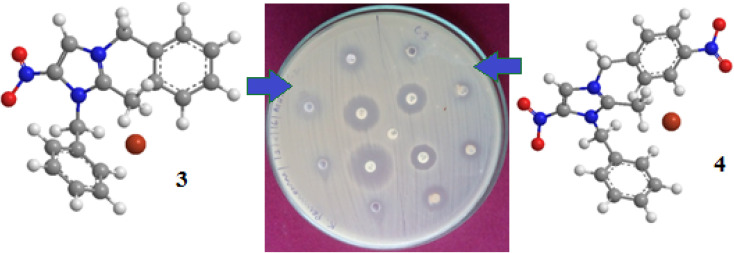
Zone of inhibition against *K. pneumoniae* with different concentrations (25, 50, 75 and 100 μg/well) of DBMNIB 3 and 4.

**Fig. 2:**
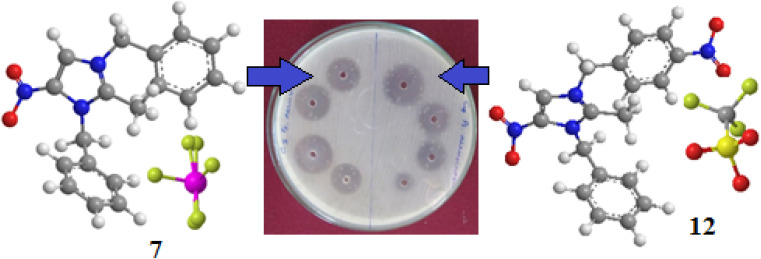
Zone of inhibition against *P. aeruginosa* with different concentrations (25, 50, 75 and 100 μg/well) of compounds 7 and 12.

The bacterial screening of more active nitro-substituted imidazolium salts against six different human pathogens was examined. Nitro-substituted imidazolium salts exhibited a greater response than other imidazolium salts. Focusing on bacterial inhibition revealed that counter anions play a crucial role in their response. Bromide-containing imidazolium salts 3 and 5 showed effective inhibition with *E. coli*, *P. aeruginosa* and *S. aureus* pathogens. Other imidazolium salts exhibited good to moderate inhibition against our test organisms, as shown in Fig. 1 and 2.

The following concentrations of imidazolium salts are used for antibacterial studies [25 μg per well, 50 μg per well, 75 μg per well and 100 μg per well]. Each well capacity is 30–50 μl. Values are measured by a Vernier caliper (diameter in mm).

### MIC/MBC determination by colorimetric method

Broth microdilution tests were performed according to NCCLS guidelines. Serial two-fold dilutions of each imidazolium salt (from an original working solution which had been 0.22 μm sterile filtered) in MHB (100 ml) were prepared in 96-well microtitre plates over the range of 10–100 μg per well. The inoculum to be tested was prepared by adjusting the turbidity of an actively overnight growing broth culture in MHB to an optical density at 550 nm equivalent to 1 × 10^−8^ CFU cm^−3^. The suspension was further diluted to give a final inoculum density of 2 × 10^−5^ CFU cm^−3^ in MHB, as verified by the total viable count. The microtitre plate for the determination of MIC and MBC was set up as described elsewhere.^57^ Positive and negative controls are included in each plate. All controls and test concentrations were prepared as six replicates. The microtitre plates were then incubated for 24 h at 37 °C. Following determination of the MIC for each compound, the MBC were derived by transferring 20 μL of the suspension from the wells, which displayed no signs of growth to MHA plates. The MHA plates were then incubated in a stationary incubator at 37 °C for 24 h and examined for 99.9% killing (Table 3).

### Docking studies

**Fig. 3:**
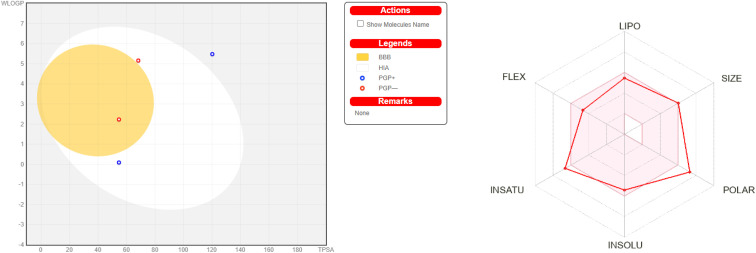
*K. pneumoniae* (5hsg) interaction with 3-NBBMNIT 8 showing the hydrogen bonding (−5.12) with an amino acid like GLN42 (−0.8078), ILE67 (−0.5322), LYS52 (−1.4266), LEU49 (−0.9565), GLN55 (−0.5375), and LEU45 (−0.4015).

**Fig. 4:**
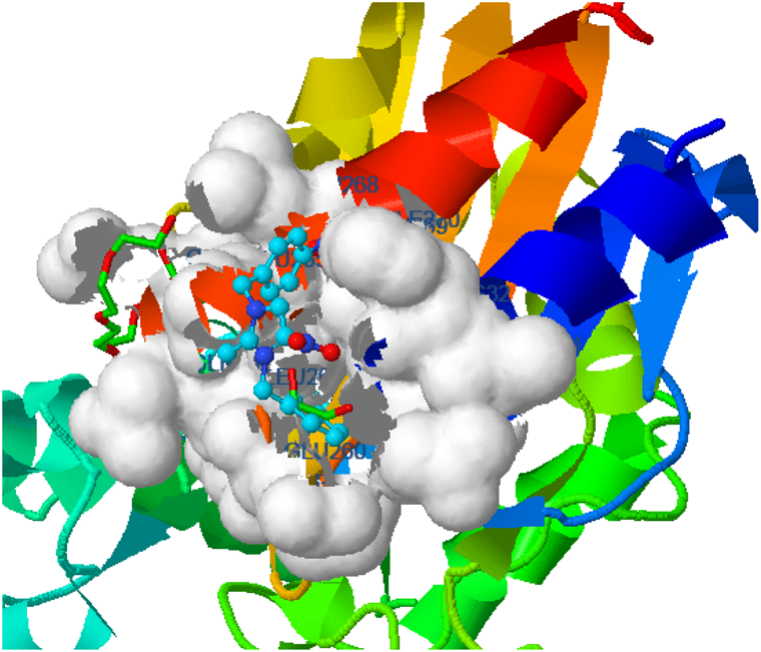
*Pseudomonas aeruginosa* (5eoe) interaction with 3-NBBMNIT 8 showing the hydrogen bonding (−5.13) with an amino acid like GLU260 (−0.7717), ASN33 (−0.7126), LEU61 (−0.4599), HIS32 (−0.4947), GLN34 (−0.7632), and GLU265 (−0.4266).

**Fig. 5:**
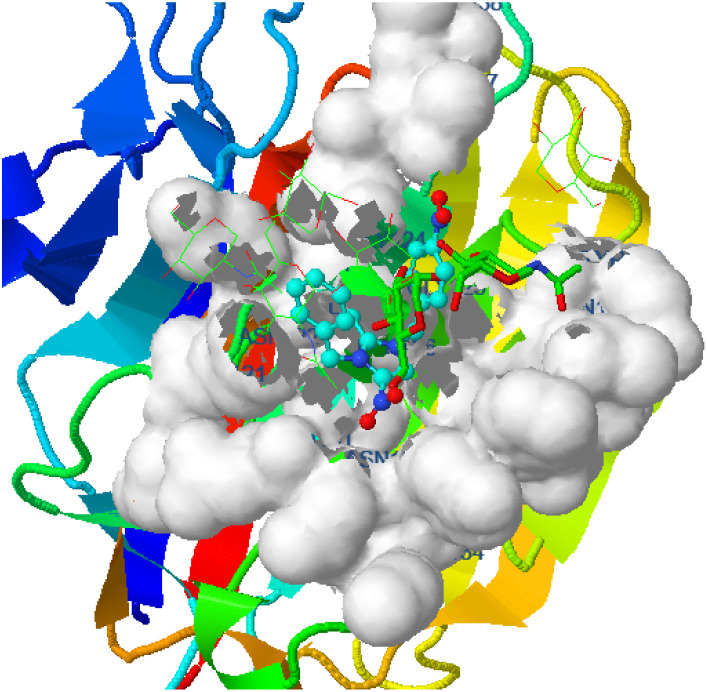
*Proteus vulgaris* (5ava) interaction with 3-NBBMNIT 8 showing the hydrogen bonding (−5.56) with an amino acid like THR237 (−0.7841), ASN151 (−0.6154), ILE236 (−2.0875), ASP107 (−0.3738), LEU149 (−0.387), and LYS123 (−0.1853).

Hydrogen bonding, ligand/nonligand bonding, and van der Waals bonding were studied using various protein sequences against the synthesized dibenzyl imidazolium salts using computer assisted docking studies. Fig. 3–5 indicate how pathogens effectively bind with dibenzyl imidazolium salts 3–18*via* host–guest interactions (ligand bond, hydrogen bonding *etc.*). Effective binding between 3-NBBMNIT 8 and *P. aeruginosa* was examined (Table 3).

*Pseudomonas aeruginosa* (5eoe) as a host for membrane protein structure determination: a comprehensive analysis of protein databases was performed using a unique membrane protein method (X-ray diffraction), with resolution (1.60 Å), crystal structure of DHFR 20% isopropanol, asymmetric-C1, monomer-A1, *R*-value free (0.167), *R*-value Work (0.142) strain K12, modeled residue count (318), total structure weight (58.85 kDa), atom count 5139, unique protein chain (1), modelled residue count (524), and deposited residue count (526) (PDB DOI: 10.2210/pdb5EOE/pdb) (Fig. 4). Six amino acids of *Pseudomonas aeruginosa* (5EOE), namely, GLN34 (A), GLU260 (A), LEU269 (A), LEU261 (A), GLU265 (A) and LEU268 (A), show effective binding with the dibenzyl imidazolium nitrogen and oxygen (Fig. 8). Among these amino acids, the nitro-substituted dibenzyl imidazolium cation with BF_4_^−^ anion 8 shows effective binding interaction such as ASN33(A) (3.36) with amino acids (Table 3 and Fig. 8).

**Fig. 6:**
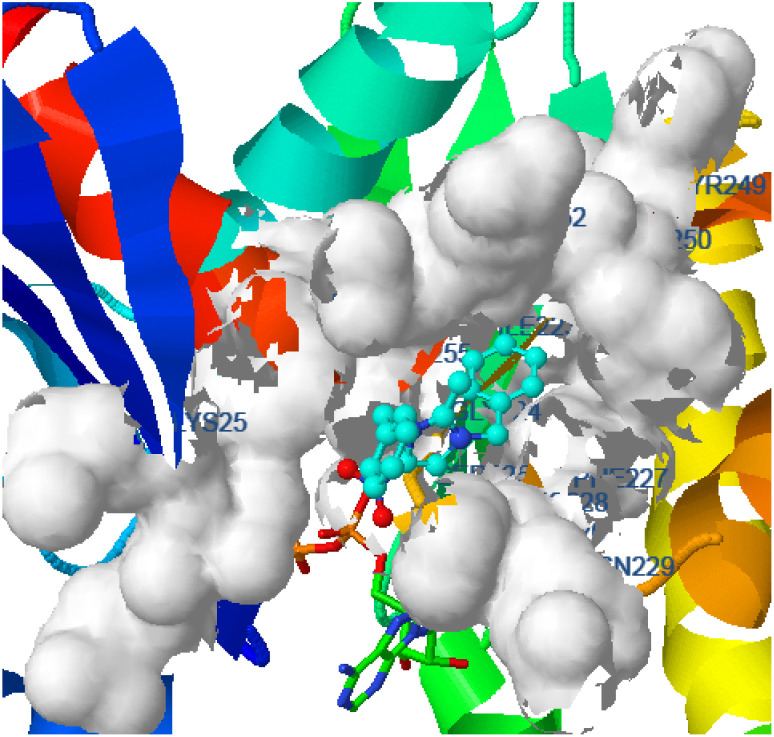
*Staphylococcus aureus* (5elz) interaction with DBMNIB 3 showing the hydrogen bonding (−6.85) with an amino acid like LYS30 (−0.7245), ASP136 (−0.4454), LEU28 (−2.1518), LEU11 (−0.8312), GLN125 (−0.4964), and THR10 (−0.3645).

**Fig. 7:**
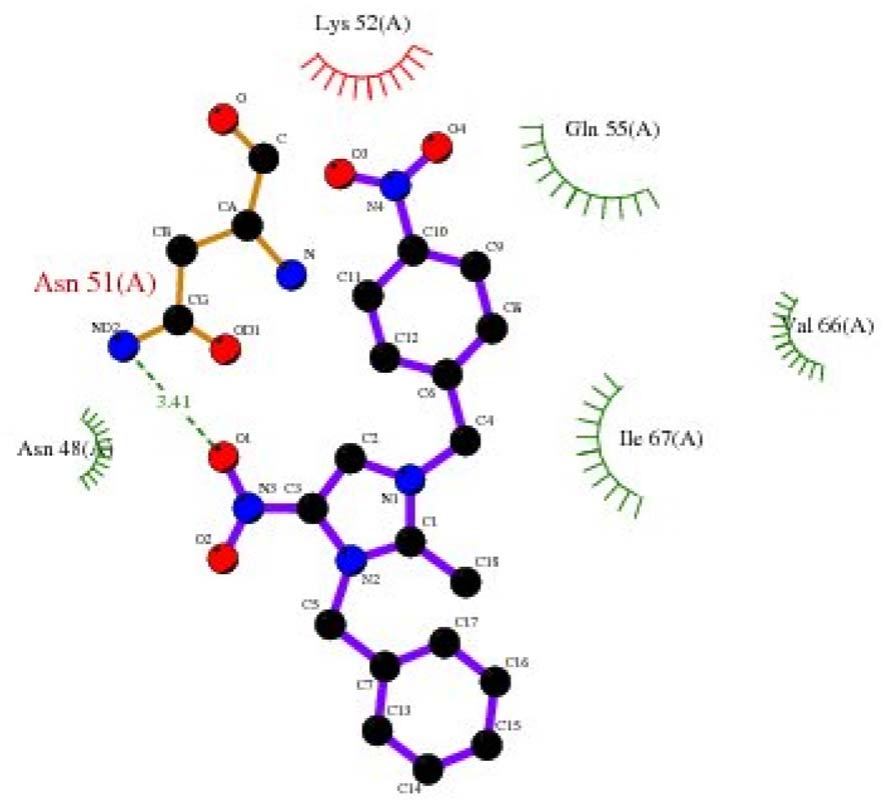
2D plot of *K. pneumoniae* (5HSG) interacting with 3-NBBMNIT 8.

**Fig. 8:**
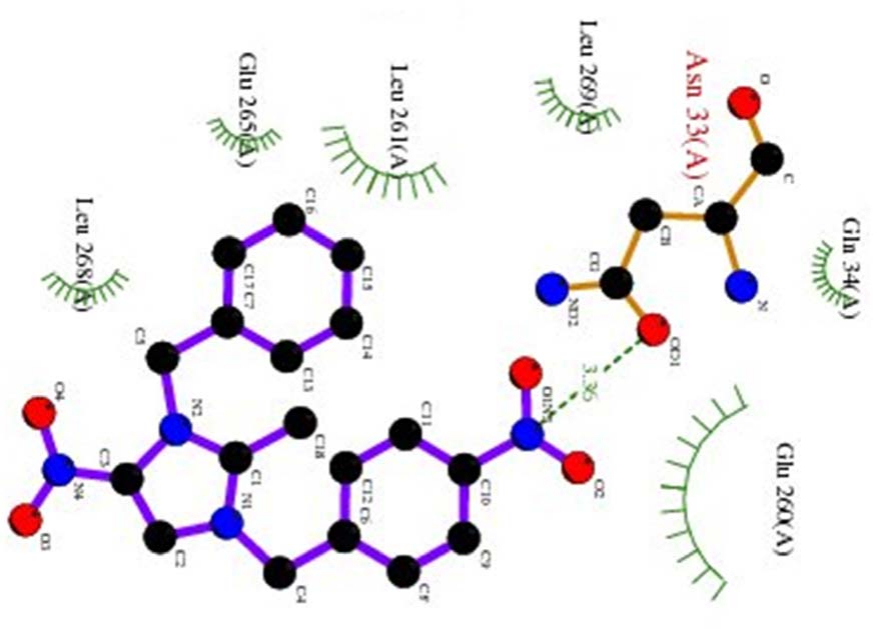
2D plot of *Pseudomonas aeruginosa* (5EOE) interacting with 3-NBBMNIT 8.

**Fig. 9:**
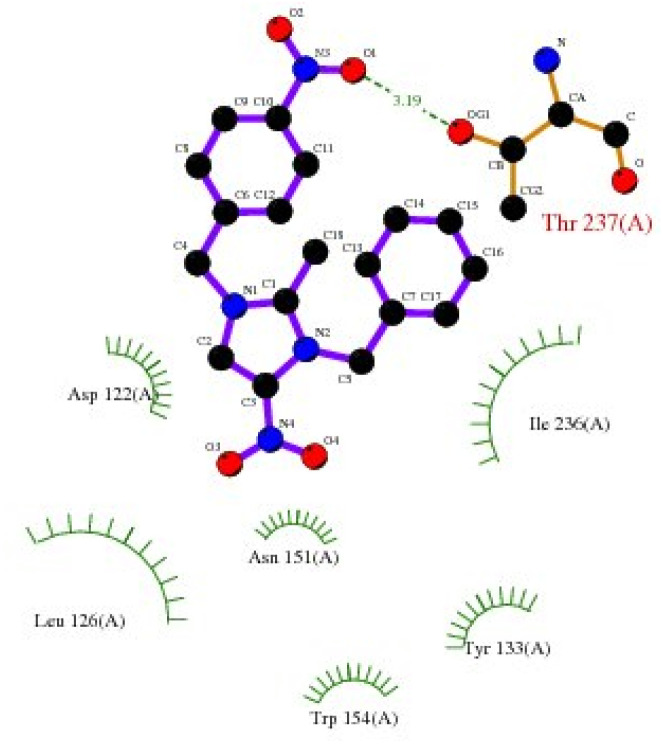
2D plot of *Proteus vulgaris* (5AVA) interacting with 3-NBBMNIT 8.

**Fig. 10:**
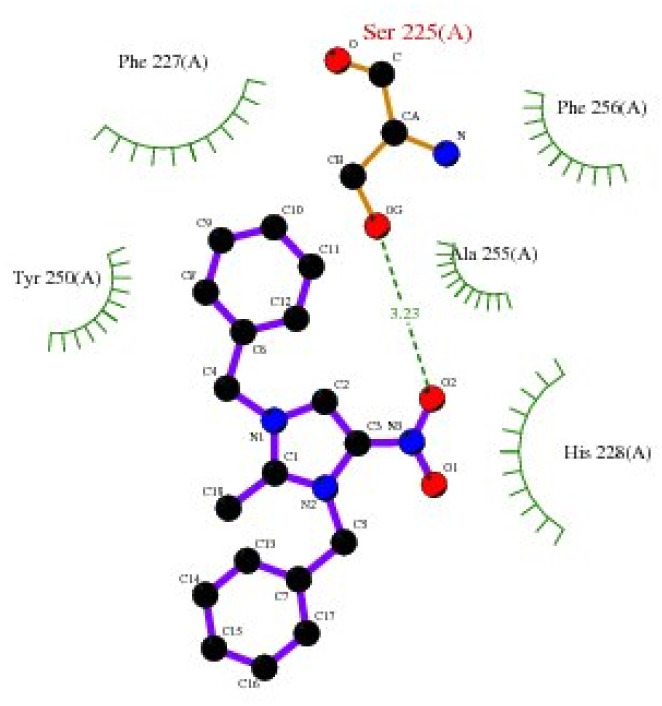
2D plot of *Staphylococcus aureus* (5ELZ) interacting with DBMNIB 3.

Bonding between the six amino acids of *Proteus vulgaris* (5AVA), namely, ASP122 (A), IIC236 (A), ASN151 (A), LEU126 (A), TYR133 (A) and TRP154 (A), and those of 3-NBBMNIT 8 such as THR237 (A) (3.19 A) with imidazolium nitrogen and oxygen (Fig. 9) was observed, whereas the nitro-substituted dibenzyl imidazolium cation with the BF_4_^−^ anion showed moderate binding with proteins (Tables 3 and 4). Hydrogen bonding studies with nitro-substituted imidazolium bromide against various pathogens like *E. coli*., *P. aeruginosa*, *P. vulgaris*, and *S. aureus* were carried out. *Staphylococcus aureus* was used as the host for the membrane protein structure determination. Among these pathogens (Fig. 3–10), the nitro-substituted dibenzyl imidazolium salts 6–8 showed the highest intermolecular binding in the order *P. aeruginosa* > *P. vulgaris* > *S. aureus* > *K. pneumoniae* > *E. coli* based on the number of hydrogen bonding, intermolecular energy, residues, and other physical parameters (Tables 3 and 4). Other dibenzyl imidazolium salts (3–5 and 9–18) have shown good to moderate values (Tables 5–8).

**Table 4:** van der Waals, hydrogen bonding electric force, and effective hydrogen bonding between the amino acids of the substituted imidazolium salts 3–7, 9, 11 and 15

Organism			Substituted dibenzyl imidazolium cation with various counter anions							
			DBMNIB 3	3-NBBMNIB 4	1-NBBMNIB 5	BNBMNIB 6	DBMNIT 7	DBMNIH 11	DBMNIT 15	3-NBBMNIT 8
*Escherichia coli* 5e8q	Est. Free energy of binding (kcal mol^−1^)		−6.39	−6.57	−6.33	−6.54	−6.68	−6.89	−7.06	−6.87
	Est. inhibition Constant, Ki (μM)		20.87	15.25	23.07	16.16	12.69	8.93	6.68	9.23
	vdW + Hbond + desolv energy		−7.69	−7.72	−7.74	−7.92	−8.11	−8.29	−8.32	−8.60
	Electrostatic energy		−0.22	−0.35	−0.35	−0.25	−0.57	−0.40	−0.47	−0.35
	Total intermolecular energy		−7.91	−8.07	−8.09	−8.16	−8.68	−8.69	−8.79	−8.96
	Interact. surface		774.222	804.661	804.745	785.496	902.549	880.1	894.188	871.327
	Decomposed interaction energies (kcal mol^−1^)	Hydrogen bond	ARG52 (−0.3338)	THR46 (−1.169)	ILE94 (−0.5281)	THR46 (−0.9443)	THR46 (−0.7003)	HIS45 (−0.3986)	ILE5 (−0.3524)	TRP22 (−18.768)
			TYR100 (0.0591)	TRP22 (−0.4649)	THR46 (−0.6081)	LEU28 (−0.5357)	SER49(−0.5461)	THR123 (-0.2711)	TRP22 (−0.9875)	THR46 (−0.4167)
		Polar	PHE31 (−1.8255)	PHE31 (−1.0741)	TRP22 (−0.7302)	PHE31 (−1.5114)	TRP22 (−1.2092)	TRP22 (−1.254)	ARG52 (−0.4181)	SER49 (−2.7207)
			LEU28 (−1.5434)	ALA7 (−0.7016)	TYR100 (−0.3679)	ILE50 (−1.0876)	PHE31 (−1.0031)	MET20 (−0.7802)	TYR100 (−0.3529)	ILE14 (−2.7356)
		Hydrophobic	SER49 (−0.3374)	ILE14 (−0.6914)	PHE31 (−0.8745)	ALA7 (−0.6536)	ASP27 (−0.4099)	THR46 (−0.7484)	ILE50 (−0.7652)	ALA19 (−2.8948)
			ASP27 (−0.2034)	SER49 (−0.47)	ILE5 (−0.5932)	SER49 (−0.4884)	LEU54 (−0.2426)	PHE31 (−0.7306)	ALA7 (−0.6829)	LEU28 (−2.3377)
*Klebsiella pneumoniae* 5hsg	Est. Free energy of binding (kcal mol^−1^)		−3.14	−2.65	−2.79	−3.18	−2.16	−2.24	−2.75	−2.75
	Est. inhibition constant, Ki (μM)		5.00	11.37	9.02	4.64	26.14	22.96	9.64	9.64
	vdW + Hbond + desolv energy		−5.56	−4.52	−4.40	−5.04	−5.15	−5.41	−5.21	−5.21
	Electrostatic energy		+0.32	+0.19	+0.06	+0.09	+0.08	+0.29	+0.14	+0.14
	Total intermolecular energy		−5.23	−4.32	−4.34	−4.96	−5.07	−5.12	−5.07	−5.07
	Interact. surface		561.99	556.181	557.143	530.582	720.025	573.314	593.946	593.946
	Decomposed interaction energies (kcal mol^−1^)	Hydrogen bond	ILE67 (−0.9302)	ASN48 (−1.0455)	ASN51 (−1.0088)	LEU49 (−0.674)	ILE67 (−0.8074)	ASN48 (−1.4162)	GLN42 (−0.8078)	GLN42 (−0.8078)
			ASN51 (−1.5049)	ILE67 (−1.019)	GLN42 (−0.5279)	ILE67 (−0.5345)	LEU315 (−0.5689)	ASN51 (−1.0761)	ILE67 (−0.5322)	ILE67 (−0.5322)
		Polar	LYS52 (−1.3393)	LYS52 (−1.3885)	LEU49 (−0.8657)	LYS52 (−1.1739)	LYS52 (−1.4042)	LYS52 (−1.3267)	LYS52 (−1.4266)	LYS52 (−1.4266)
			GLN55 (−0.8016)	ASN51 (−0.9546)	LEU315 (−0.1067)	ASN51 (−1.1598)	ASN48 (−0.9324)	GLN55 (−0.9456)	LEU49 (−0.9565)	LEU49 (−0.9565)
		Hydrophobic	ASN48 (−0.7729)	GLN42 (−0.5656)	LYS52 (−1.5395)	ASN48 (−1.141)	GLN42 (−0.7446)	GLN42 (−0.7817)	GLN55 (−0.5375)	GLN55 (−0.5375)
			LEU49 (−0.5812)	ILE67 (−0.2009)	ASN48 (−0.9427)	GLN42 (−0.8885)	LEU49 (−0.6654)	LEU49 (−0.4413)	LEU45 (−0.4015)	LEU45 (−0.4015)
*Pseudomonas aeruginosa* 5eoe	Est. Free energy of binding (kcal mol^−1^)		−4.33	−4.32	−3.39	−4.19	−4.19	−4.44	−3.92	−4.15
	Est. inhibition constant, Ki (μM)		666.66	677.81	3.29	844.62	844.62	558.30	1.33	909.93
	vdW + Hbond + desolv energy		−5.34	−4.50	−4.04	−5.13	−5.13	−5.52	−5.14	−5.13
	Electrostatic energy		−0.93	−0.67	−0.86	−0.81	−0.81	−0.90	−0.76	−0.85
	Total intermolecular energy		−6.27	−5.17	−4.90	−5.94	−5.94	−6.42	−5.89	−5.98
	Interact. surface		547.484	498.744	388.931	555.689	555.689	531.433	533.07	525.241
	Decomposed interaction energies	Hydrogen bond	GLU260 (−0.2127)	GLU260 (−1.1649)	GLU260 (−1.0302)	GLU265 (−0.8307)	GLU265 (−0.8307)	GLU260 (−4.7237)	GLU260 (−1.5775)	GLU260 (−0.7717)
			GLU265 (−0.444)	GLN34 (−0.3613)					GLU265 (−0.2351)	ASN33 (−0.7126)
		Polar	ASN33 (−1.2409)	LEU261 (−0.8181)	GLN34 (−1.3633)	ASN33 (−1.0306)	ASN33 (−1.0306)	HIS32 (−1.6994)	LEU261 (−0.4844)	LEU261 (−0.4599)
			LEU268 (−0.1727)	HIS32 (−0.5389)		GLN262 (−0.6469)	GLN262 (−0.6469)			HIS32 (−0.4947)
		Hydrophobic	LEU261 (−0.5892)	GLU265 (−0.5724)	LEU261 (−0.3147)	LEU261 (−1.113)	LEU261 (−1.113)	GLU265 (−0.2677)	GLN34 (−0.3402)	GLN34 (−0.7632)
			HIS32 (−0.1266)	ASN33 (−0.4484)						GLU265 (−0.4266)
*Proteus vulgaris* 5ava	Est. free energy of binding (kcal mol^−1^)		−3.80	−4.41	−4.47	−4.70	−3.91	−4.77	−3.93	−4.16
	Est. inhibition constant, Ki (μM)		1.63	586.97	528.96	360.63	1.35	321.39	1.32	889.77
	vdW + Hbond + desolv energy		−5.00	−5.71	−6.09	−5.67	−5.78	−5.90	−5.63	−5.56
	Electrostatic energy		−0.35	−0.22	−0.24	−0.70	−0.09	−0.82	−0.24	−0.44
	Total intermolecular energy		−5.35	−5.93	−6.33	−6.37	−5.87	−6.73	−5.87	−6.00
	Interact. surface		651.368	529.682	638.71	646.428	540.796	641.566	694.239	625.594
	Decomposed interaction energies (kcal mol^−1^)	Hydrogen bond	ASN151 (−0.9592)	LYS123 (−0.3095)	ILE236 (−5.9305)	ASN151 (−0.5675)	HIS153 (−1.11)	ASP122 (−0.857)	ASN151 (−1.3023)	THR237 (−0.7841)
			LEU149 (−0.635)	ASN151 (−0.4697)	HIS153 (−0.8931)	ASP122 (−0.4816)	THR237 (−0.3305)	SER65 (−0.3013)	ASP122 (−0.2392)	ASN151 (−0.6154)
		Polar	HIS153 (−0.8232)	THR237 (−0.4749)	ASN151 (−0.8103)	ILE236 (−1.3359)	ASN240 (−0.3097)	ILE236 (−1.2928)	ILE236 (−0.8975)	ILE236 (−2.0875)
			TRP154 (−0.7822)	ASP107 (−0.4723)	LEU126 (−0.5207)	LEU126 (−0.511)	VAL152 (−1.3751)	LYS123 (−0.2438)	HIS153 (−0.5475)	ASP107 (−0.3738)
		Hydrophobic	TYR133 (−0.5787)	ILE236 (−1.6483)	TYR133 (−0.5082)	LYS123 (−0.5801)	ASN151 (−1.0309)	ASN151 (−2.4336)	TYR150 (−0.5109)	LEU149 (−0.387)
			THR237 (−0.5329)	LEU149 (−0.4689)	VAL152 (−0.0905)	ASP107 (−0.3125)	LEU149 (−0.751)	LEU126 (−0.5397)	TRP154 (−0.4859)	LYS123 (−0.1853)
*Staphylococcus aureus* 5elz	Est. free energy of binding (kcal mol^−1^)		−5.83	−5.67	−5.46	−5.52	−6.38	−5.81	−4.78	−6.26
	Est. inhibition constant, Ki (μM)		53.73	69.53	100.09	89.84	20.99	55.04	312.33	25.88
	vdW + Hbond + desolv energy		−7.89	−7.91	−7.28	−7.04	−8.40	−7.80	−6.85	−8.09
	Electrostatic energy		−0.06	+0.13	−0.03	−0.03	−0.06	+0.06	−0.04	−0.26
	Total intermolecular energy		−7.95	−7.78	−7.31	−7.07	−8.46	−7.74	−6.89	−8.36
	Interact. surface		773.952	711.001	754.301	758.146	801.719	720.589	754.464	806.736
	Decomposed interaction energies (kcal mol^−1^)	Hydrogen bond	GLN125 (−0.9321)	HIS228 (−2.3776)	TYR137 (−1.9398)	HIS228 (−0.6751)	TYR137 (−2.6511)	HIS228 (−2.2299)	LYS30 (−0.7245)	GLY100 (−0.7251)
			HIS228 (−0.8384)	THR26 (−0.6981)	HIS228 (−1.0564)	ASP136 (−0.4283)	HIS228 (−2.0876)	THR26 (−1.1699)	ASP136 (−0.4454)	GLU70 (−0.5634)
		Polar	TYR137 (−2.2474)	PHE256 (−0.9752)	LYS138 (−0.7523)	TYR137 (−2.5512)	LEU11 (−1.1549)	PHE256 (−0.6929)	LEU28 (−2.1518)	THR99 (−1.4395)
			LEU11 (−1.1795)	PHE227 (−0.8065)	GLN125 (−0.7265)	LEU11 (−1.1817)	THR26 (−0.916)	LEU28 (−0.5639)	LEU11 (−0.8312)	ASN96 (−0.7876)
		Hydrophobic	THR10 (−0.909)	TYR250 (−0.702)	LEU11 (−0.8865)	LYS138 (−0.6714)	LYS138 (−0.6196)	SER225 (−0.5015)	GLN125 (−0.4964)	THR10 (−0.7291)
			LYS138 (−0.5646)	SER225 (−0.6341)	THR10 (−0.5822)	THR10 (−0.6449)	PHE256 (−0.3863)	PHE227 (−0.4049)	THR10 (−0.3645)	SER225 (−0.5945)

**Table 5:** Van der Waals, hydrogen bonding electric force, and effective hydrogen bonding between amino acids of substituted imidazolium salts 9, 10, 12–14, 16–18

Organism			Substituted dibenzyl imidazolium cation with various counter anions							
			3-NBBMNIH 12	3-NBBMNIT 16	1-NBBMNIT 9	1-NBBMNIH 13	1-NBBMNIH 17	BNBMNIT 10	BNBMNIH 14	BNBMNIT 18
*Escherichia coli* 5e8q	Est. free energy of binding (kcal mol^−1^)		−6.41	−7.05	−6.77	−7.70	−6.81	−6.86	−6.45	−6.67
	Est. inhibition constant, Ki (μM)		20.11	6.81	10.83	2.27	10.24	9.35	18.84	13.00
	vdW + Hbond + desolv energy		−7.37	−8.05	−8.02	−8.70	−7.32	−7.50	−7.25	−7.33
	Electrostatic energy		−0.33	−0.27	−0.27	−0.39	−0.53	−0.38	−0.24	−0.39
	Total intermolec. energy		−7.70	−8.33	−8.29	−9.08	−7.85	−7.87	−7.50	−7.72
	Interact. surface		791.08	781.028	810.513	783.169	737.317	739.736	779.384	753.466
	Decomposed interaction energies (kcal mol^−1^)	Hydrogen bond	ILE94 (−0.5392)	ARG57 (−0.6096)	SER49 (−0.3975)	TRP22 (−0.9556)	PHE31 (−1.9902)	PHE31 (−2.0224)	ASP27 (−0.2119)	PHE31 (−1.3063)
			ARG52 (−0.4847)	PHE31 (−1.7537)	ASP27 (−0.3388)	PHE31 (−0.9682)	ILE14 (−0.8003)	MET20 (−0.5924)	PHE31 (−1.327)	ILE5 (−0.5966)
		Polar	ASP27 (−0.4035)	LEU28 (−1.1077)	PHE31 (−1.085)	LEU28 (−0.8389)	LEU28 (−0.4351)	ILE94 (−0.748)	ILE5 (−0.6223)	MET20 (−0.3131)
			THR46 (−0.3793)	ILE50 (−0.7601)	ILE14 (−0.6789)	MET20 (−0.7528)	TYR100 (−0.7731)	TYR100 (−0.5227)	LEU28 (−0.4127)	TRP22 (−0.6794)
		Hydrophobic	LYS32 (−0.3695)	ILE5 (−0.6027)	LEU28 (−0.6222)	ILE94 (−0.5322)	TRP22 (−0.2805)	ASP27 (−0.2989)	ALA7 (−0.5787)	ILE94 (−0.6136)
			TYR100 (−0.557)	LEU54 (−0.5733)	ALA7 (−0.5931)	LEU24 (−0.5055)	MET20 (−0.2596)	ILE50 (−0.2405)	THR46 (−0.4594)	THR46 (−0.4504)
*Klebsiella pneumoniae* 5hsg	Est. free energy of binding (kcal mol^−1^)		−3.60	−3.61	−3.46	−3.46	−3.12	−3.28	−2.76	−3.57
	Est. inhibition constant, Ki (μM)		2.30	2.26	2.90	2.90	5.15	3.93	9.41	2.41
	vdW + Hbond + desolv energy		−5.48	−5.05	−5.08	−5.08	−4.47	−4.80	−4.04	−4.70
	Electrostatic energy		+0.24	+0.21	+0.09	+0.09	+0.22	+0.12	+0.08	+0.09
	Total intermolecular energy		−5.24	−4.83	−4.98	−4.98	−4.24	−4.68	−3.97	−4.61
	Interact. surface		566.743	553.356	554.694	554.694	519.265	529.467	514.644	510.986
	Decomposed interaction energies (kcal mol^−1^)	Hydrogen bond	ASP56 (−0.2705)	ASN51 (−2.0002)	ASN51 (−1.117)	ASN51 (−1.117)	GLN42 (−0.4792)	ASN51 (−1.4079)	LEU49 (−0.5151)	LEU49 (−0.6909)
			ASN51 (−0.7004)	ASN48 (−0.1847)	GLN42 (−0.2573)	GLN42 (−0.2573)	LYS52 (−0.9766)	ASN48 (−0.9078)	LEU315 (−0.3332)	ILE67 (−0.6298)
		Polar	ILE67 (−0.4889)	ILE67 (−0.9451)	LEU315 (−0.6494)	LEU315 (−0.6494)	ILE67 (−1.1879)	GLN42 (−0.5289)	LYS52 (−1.571)	ASN51 (−1.7298)
			LEU49 (−0.1117)	VAL66 (−0.3368)	LEU49 (−0.452)	LEU49 (−0.452)	ASN51 (−2.7041)	LEU49 (−0.6046)	ASN51 (−0.8454)	LYS52 (−1.075)
		Hydrophobic	ASN48 (−3.4384)	GLN55 (−1.1263)	LYS52 (−2.4038)	LYS52 (−2.4038)	GLN55 (−0.4273)	ILE67 (−0.4227)	ASN48 (−0.7206)	GLN42 (−0.509)
			GLN55 (−1.1746)	ASN132 (−0.1923)	ASN48 (−0.8138)	ASN48 (−0.8138)	LYS52 (−1.2024)	GLN55 (−1.3029)	GLN42 (−0.6852)	GLN55 (−0.3918)
*Pseudomonas aeruginosa* 5eoe	Est. free energy of binding (kcal mol^−1^)		−4.69	−4.93	−4.09	−5.39	−3.77	−3.79	−3.37	−4.32
	Est. inhibition constant, Ki (μM)		364.66	242.57	1.01	112.15	1.74	1.66	3.37	677.81
	vdW + Hbond + desolv energy		−5.27	−5.56	−4.70	−5.73	−4.40	−3.73	−3.61	−4.50
	Electrostatic energy		−0.85	−0.74	−0.70	−0.88	−0.76	−1.05	−0.81	−0.67
	Total intermolecular energy		−6.13	−6.30	−5.40	−6.61	−5.16	−4.77	−4.42	−5.17
	Interact. surface		531.548	553.955	511.214	588.294	483.968	451.098	403.645	498.744
	Decomposed interaction energies	Hydrogen bond	GLU260 (−1.205)	GLU265 (−1.3363)	ASN33 (−0.7332)	ASN33 (3.5619)	GLU260 (−0.9751)	GLU260 (−0.849)	GLU260 (−0.2495)	GLU260 (−1.1649)
			PRO259 (−0.4366)	ASN33 (−0.65)	GLU265 (−0.7168)	GLU260 (−3.0931)	GLU265 (−0.2799)	GLU265 (−0.1502)	TYR100 (−0.4024)	LEU261 (−0.8181)
		Polar	GLN262 (−0.7342)	LEU261 (−1.0896)	GLN34 (−0.8774)	LEU261 (−1.1655)	LEU261 (−0.7915)	GLN262 (−0.7601)	LEU261 (−1.8244)	HIS32 (−0.5389)
			LEU261 (−0.7594)	GLU260 (−0.5957)	GLU260 (−0.7479)	LEU269 (−0.421)	ASP122 (−0.3768)	HIS153 (−0.3539)	ILE50 (−0.2927)	GLU265 (−0.5724)
		Hydrophobic	HIS32 (−0.1527)	LEU269 (−0.247)	LEU261 (−0.7462)	LEU268 (−0.3055)	GLN262 (−0.5791)	LEU261 (−0.5132)	ASP27 (−0.018)	ASN33 (−0.4484)
			ASN33 (−0.758)	LEU268 (−0.1217)	GLN262 (−0.6878)	GLU265 (−0.1064)	TRP154 (−0.7364)	ASP122 (−0.2935)	ASN48 (−0.3523)	GLN34 (−0.3613)
*Proteus vulgaris* 5ava	Est. free energy of binding (kcal mol^−1^)		−3.94	−3.88	−4.50	−4.07	−4.44	−4.95	−4.72	−4.86
	Est. inhibition constant, Ki (μM)		1.28	1.42	500.28	1.05	557.18	236.94	348.31	276.10
	vdW + Hbond + desolv energy		−5.29	−4.90	−5.36	−4.98	−5.26	−5.95	−5.40	−5.41
	Electrostatic energy		−0.17	−0.47	−0.53	−0.47	−0.30	−0.32	−0.39	−0.38
	Total intermolecular energy		−5.46	−5.36	−5.90	−5.45	−5.56	−6.26	−5.79	−5.79
	Interact. Surface		618.237	664.576	632.176	583.171	625.816	635.278	643.154	537.171
	Decomposed interaction energies (kcal mol^−1^)	Hydrogen bond	ASN151 (−0.9426)	ILE236 (−0.7708)	THR237 (−0.2745)	HIS153 (−0.5526)	ASN151 (−1.1376)	ASN151 (−0.5414)	THR237 (−0.349)	TRP154 (−5.0427)
			LEU126 (−0.6151)	TRP154 (−0.711)	TRP154 (−0.3873)	ASP122 (−0.3659)	ASP107 (−0.3127)	ILE236 (−0.7835)	ASN151 (−0.8159)	ILE236 (−0.5225)
		Polar	ILE236 (−0.9482)	TYR133 (−0.7044)	LEU126 (−1.5408)	LEU126 (−1.8945)	LEU126 (−1.1293)	TRP154 (−0.7191)	LEU149 (−0.9831)	HIS153 (−0.0522)
			VAL152 (−0.4984)	LEU149 (−0.4546)	ILE236 (−1.3614)	TRP154 (−0.6094)	ILE236 (−0.9123)	LEU126 (−0.574)	ASP107 (−0.4653)	ASP122 (−0.0956)
		Hydrophobic	LEU149 (−0.1978)	ASN151 (−0.984)	TYR133 (−0.4903)	TYR133 (−0.4673)	TRP154 (−0.6364)	LEU149 (−0.488)	ILE236 (−0.8859)	TYR133 (0.0356)
			ASP122 (−0.0678)	ASP107 (−0.2788)	ASP122 (−0.4514)	ASN151 (−0.3677)	EU149 (−0.4056)	ASN240 (−0.3759)	ASP122 (−0.3273)	LEU126 (0.3113)
*Staphylococcus aureus* 5elz	Est. free energy of binding (kcal mol^−1^)		−4.06	−5.05	−6.09	−6.41	−6.32	−5.30	−6.07	−4.85
	Est. inhibition constant, Ki (μM)		1.05	197.73	34.30	19.94	23.19	131.26	35.50	277.07
	vdW + Hbond + desolv energy		−5.39	−6.34	−7.92	−7.56	−7.71	−6.46	−7.48	−5.91
	Electrostatic energy		−0.95	−0.06	+0.02	−0.11	−0.03	−0.13	+0.11	+0.06
	Total intermolecular energy		−6.34	−6.40	−7.91	−7.66	−7.75	−6.58	−7.38	−5.85
	Interact. Surface		637.534	655.526	747.011	779.791	753.327	668.932	639.113	689.763
	Decomposed interaction energies (kcal mol^−1^)	Hydrogen bond	LEU11 (−1.032)	TYR137 (−2.2352)	GLN125 (−0.946)	SER225 (−0.8248)	HIS228 (−1.0915)	THR26 (−0.2963)	SER225 (−0.6281)	SER225 (−0.3394)
			LEU28 (−0.7031)	THR26 (−0.7303)	THR26 (−0.4318)	HIS228 (−0.6544)	TYR137 (−2.6077)	TYR137 (−2.3192)	HIS228 (−1.2723)	HIS228 (−0.6861)
		Polar	ASN151 (−0.2713)	LEU11 (−1.2483)	GLU27 (−0.4651)	THR99 (−0.4289)	LEU11 (−0.7823)	LEU28 (−1.0047)	PHE227 (−1.2129)	TYR137 (−1.2845)
			HIS188 (−0.6742)	HIS228 (−0.9501)	TYR137 (−3.0111)	LEU11 (−0.6802)	SER225 (−0.9638)	HIS228 (−0.8835)	PHE256 (−0.7219)	LEU11 (−0.5608)
		Hydrophobic	THR10 (−0.3555)	LEU28 (−0.8847)	SER226 (−0.2644)	TYR137 (−1.7021)	THR10 (−0.6077)	LEU11 (−0.7247)	TYR250 (−0.6404)	THR26 (−0.3275)
			SER226 (−0.1291)	SER225 (−0.4098)	THR10 (−0.2251)	LYS138 (−0.5825)	GLN125 (−0.5219)	GLU27 (−0.8806)	ALA255 (−0.3466)	GLN125 (−0.2254)

**Table 6:** Physicochemical properties, lipophilicity and water solubility studies

Compound	Physicochemical properties					Lipophilicity			Water solubility		
	MW (g mol^−1^)	No. heavy atoms	No. atom. heavy atoms	Molar refractivity	TPSA	Log *P* o/w (iLOGP)	Log *P* o/w (XLOGP3)	Log *P* o/w (MLOGP)	Log *S* (ESOL)	Solubility mg ml; mol l^−1^	Class
DBMNIB 3	388.26	24	17	100.97	54.63	−4.20	4.87	3.30	−5.51	1.20 × 10^−3^; 3.09 × 10^−6^	Moderately soluble
3-NBBMNIB 4	433.26	27	17	109.80	100.45	−4.84	4.70	2.38	−5.56	1.20 × 10^−3^; 2.77 × 10^−6^	Moderately soluble
1-NBBMNIB 5	433.26	27	17	109.80	100.45	−4.61	4.70	2.38	−5.56	1.20 × 10^−3^; 2.77 × 10^−6^	Moderately soluble
BNBMNIB 6	478.25	30	17	118.62	146.27	−5.06	4.52	1.55	−5.61	1.17 × 10^−3^; 2.45 × 10^−6^	Moderately soluble
DBMNIT 7	395.16	28	17	102.23	54.63	0.00	6.12	3.41	−6.26	2.15 × 10^−4^; 5.43 × 10^−7^	Poorly soluble
3-NBBMNIT 8	453.32	30	17	105.62	68.22	0.00	7.11	3.63	−7.22	2.74 × 10^−5^; 6.04 × 10^−8^	Poorly soluble
1-NBBMNIT 9	457.42	31	17	107.84	120.21	−3.88	4.22	2.60	−5.34	2.07 × 10^−3^; 4.52 × 10^−6^	Moderately soluble
BNBMNIT 10	440.16	31	17	111.05	100.45	0.00	5.95	2.49	−6.33	2.07 × 10^−4^; 4.71 × 10^−7^	Poorly soluble
DBMNIH 11	498.32	33	17	114.44	114.04	0.00	6.94	2.71	−7.29	2.57 × 10^−5^; 5.16 × 10^−8^	Poorly soluble
3-NBBMNIH 12	502.42	34	17	116.67	166.03	−4.74	4.05	1.77	−5.41	1.93 × 10^−3^; 3.85 × 10^−6^	Moderately soluble
1-NBBMNIH 13	440.16	31	17	111.06	100.45	0.00	5.95	2.49	−6.33	2.07 × 10^−4^; 4.71 × 10^−7^	Poorly soluble
BNBMNIH 14	498.32	33	17	114.44	114.04	0.00	6.94	2.76	−7.29	2.57 × 10^−5^; 5.16 × 10^−8^	Poorly soluble
DBMNIT 15	502.42	34	17	116.67	166.03	−4.98	4.05	1.77	−5.41	1.93 × 10^−3^; 3.85 × 10^−6^	Moderately soluble
3-NBBMNIT 16	485.15	34	17	119.88	146.27	0.00	5.77	3.27	−5.76	2.43 × 10^−3^; 4.57 × 10^−6^	Moderately soluble
1-NBBMNIH 17	543.31	36	17	123.26	159.86	0.00	6.77	1.88	−7.36	2.37 × 10^−5^; 4.35 × 10^−8^	Poorly soluble
BNBMNIT 18	547.42	37	17	125.49	211.85	−5.74	3.88	5.30	−5.49	0.77 × 10^−3^; 3.23 × 10^−6^	Moderately soluble

**Table 7:** Pharmacokinetic studies

Compound	GI absorption	BBB permeant	P-gp substrate	Log Kp skin permeation (cm s^−1^)	CYP2C19 inhibitor	CYP1A2 inhibitor
DBMNIB 3	High	No	Yes	−5.21	No	No
3-NBBMNIB 4	High	No	Yes	−5.61	No	Yes
1-NBBMNIB 5	High	No	Yes	−5.61	No	Yes
BNBMNIB 6	High	Yes	No	−4.37	Yes	No
DBMNIT 7	High	No	No	−4.02	Yes	No
3-NBBMNIT 8	Low	No	Yes	−6.09	Yes	No
1-NBBMNIT 9	High	No	No	−4.76	Yes	No
BNBMNIT 10	Low	No	No	−4.41	Yes	No
DBMNIH 11	Low	No	Yes	−6.49	Yes	No
3-NBBMNIH 12	Low	No	Yes	−6.5	Yes	No
1-NBBMNIH 13	High	No	No	−4.76	Yes	No
BNBMNIH 14	Low	No	No	−4.41	Yes	No
DBMNIT 15	Low	No	Yes	−6.49	Yes	No
3-NBBMNIT 16	High	No	Yes	−5.76	Yes	No
1-NBBMNIH 17	Low	No	Yes	−4.81	Yes	No
BNBMNIT 18	Low	No	Yes	−6.88	Yes	No

**Table 8:** Drug likeness and medicinal chemistry studies

Compounds	Drug likeness						Medicinal chemistry	
	Lipinski	Ghose	Veber	Egan	Muegge	Bioavailability score	Brenk	Synthetic accessibility
DBMNIB 3	Yes	Yes	Yes	Yes	Yes	0.55	3 alert	2.94
3-NBBMNIB 4	Yes	Yes	Yes	Yes	Yes	0.55	3 alert	3.04
1-NBBMNIB 5	Yes	Yes	Yes	Yes	Yes	0.55	3 alert	3.04
BNBMNIB 6	Yes	Yes	Yes	Yes	Yes	0.55	3 alert	3.04
DBMNIT 7	Yes	Yes	Yes	Yes	Yes	0.55	4 alert	3.18
3-NBBMNIT 8	Yes	Yes	Yes	Yes	Yes	0.55	4alert	3.20
1-NBBMNIT 9	Yes	Yes	Yes	Yes	Yes	0.55	4 alert	3.14
BNBMNIT 10	Yes	No	Yes	Yes	Yes	0.55	4 alert	3.28
DBMNIH 11	Yes	No	Yes	Yes	Yes	0.55	4 alert	3.25
3-NBBMNIH 12	No: 2 violations	No: 1 violation	No: 1 violation	No: 1 violation	No: 1 violation	0.55	5 alert	3.30
1-NBBMNIH 13	Yes	Yes	Yes	Yes	Yes	0.55	4 alert	3.14
BNBMNIH 14	Yes	No	Yes	Yes	No	0.55	4 alert	3.28
DBMNIT 15	No: 2 violations	No: 1 violation	No: 1 violation	No: 1 violation	No: 1 violation	0.55	5 alert	3.30
3-NBBMNIT 16	Yes	Yes	Yes	Yes	Yes	0.55	5 alert	3.26
1-NBBMNIH 17	No	No	No	No	No	0.17	4 alert	3.36
BNBMNIT 18	No: 2 violations	No: 1 violation	No: 1 violation	No: 1 violation	No: 1 violation	0.17	5 alert	3.37

### Molecular docking analysis

**Fig. 11:**
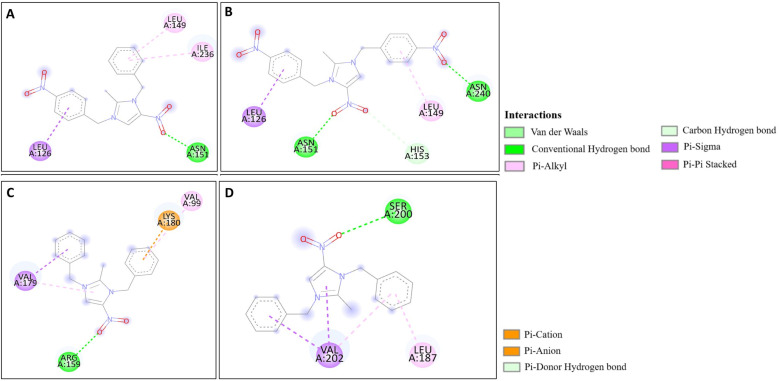
Molecular docking poses of the synthesized compounds with the 5AVA protein: (A) 3-NBBMNIB 4 forming π-alkyl interactions with LEU149 and ILE236, π-sigma interactions with LEU126, and hydrogen bonds with ASN151; (B) BNBMNIB 6 showing a π-sigma interaction with LEU126, carbon–hydrogen bond with HIS153, and hydrogen bonds with ASN151 and ASN240; (C) DBMNIB 3 forming hydrogen bonds with ARG159, π-sigma with VAL179, and π-alkyl with VAL99; and (D) DBMNIH 11 forming hydrogen bonds with SER200, π-sigma interactions with VAL202, and π-alkyl interaction with LEU187.

**Fig. 12:**
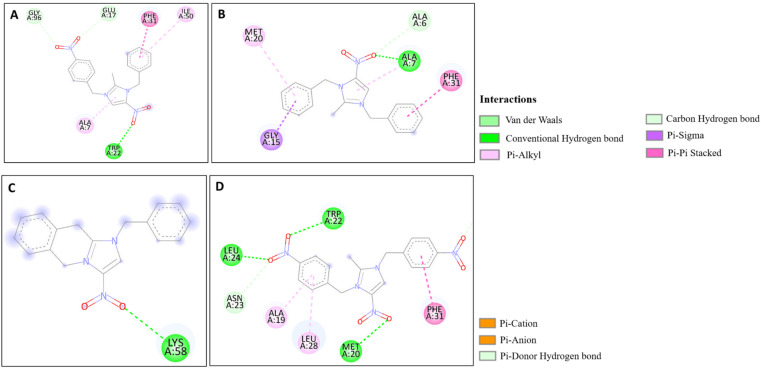
Molecular docking poses of the synthesized compounds with the 5E8Q protein: (A) 3-NBBMNIB 4 forming a H-bond with TRP22, π–π stacking with PHE31, and π-alkyl interactions with ILE50 and ALA7; (B) DBMNIH 11 displaying H-bonding with ALA7 and interactions with MET20 and PHE31; (C) DBMNIB 3 forming a H-bond with LYS58. (D) BNBMNIB 6 showing H-bonds with MET20, LEU24, and TRP22.

**Fig. 13:**
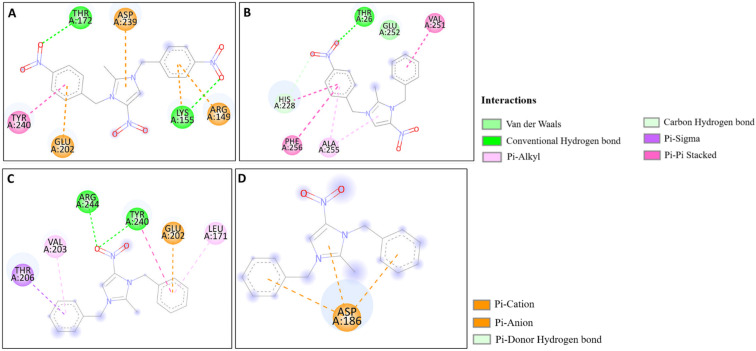
Molecular docking poses of the synthesized compounds with the 5ELZ protein: (A) BNBMNIB 6 forming hydrogen bonds with LYS155 and THR172, π-anion interaction with GLU202, and π-cation interaction with ASP239. (B) 3-NBBMNIB 4 forming π–π stacked interactions with PHE256 and HIS228. (C) DBMNIB 3 interacting with TYR240 and ARG244. (D) DBMNIH 11 exhibiting π-anion interactions with ASP186.

**Fig. 14:**
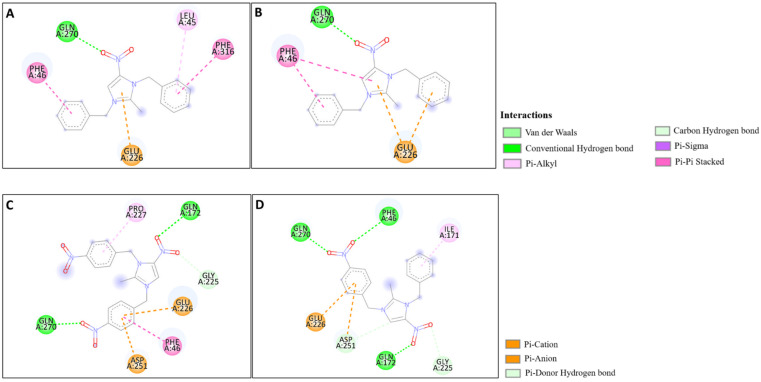
Molecular docking poses of the synthesized compounds with the 5EOE protein: (A) DBMNIH 11 forming H-bonds with LYS36 and π-cation interactions with ARG57. (B) 3-NBBMNIB 4 forming H-bonds with ARG57 and ASN257, and π-anion interaction with GLU170. (C) BNBMNIB 6 forming H-bonds with ARG57 and π-cation interaction with LYS36. (D) DBMNIB 3 interacting with LYS36 and ASN257.

**Fig. 15:**
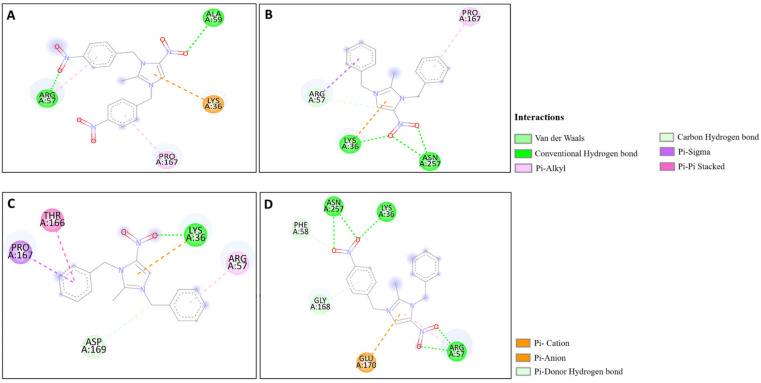
Molecular docking poses of the synthesized compounds with the 5HSG protein: (A) BNBMNIB 6 forming H-bonds with GLN270 and GLN172, π-anion interactions with ASP251 and GLU226. (B) DBMNIB 3 forming H-bonding with GLN270 and π–π stacking with PHE46. (C) 3-NBBMNIB 4 interacting with GLN270, GLN172, and GLU226. (D) DBMNIH 11 displaying H-bonding with GLN270 and π–π stacking with PHE46.

The docking studies of the synthesized compounds with bacterial pathogenic proteins showed binding interactions, indicating their potential for further investigation. Regarding the protein Vulgaris 5AVA, 3-NBBMNIB 4 (Fig. 11A) obtains the highest binding affinity scoring of −8.2 kcal mol^−1^ with π-alkyl interactions with LEU149 and ILE236; π-sigma interactions with LEU126; and hydrogen bonds with ASN151. BNBMNIB 6 (Fig. 11B) shows a π-sigma interaction with LEU126, a carbon–hydrogen bond with HIS153, and hydrogen bonds with ASN151 and ASN240, with a score of −7.9 kcal mol^−1^. DBMNIH 11 (−7.5 kcal mol^−1^) forms a hydrogen bond with SER200, π-sigma interactions with VAL202, and Π-alkyl interaction with LEU187 (Fig. 11D), while DBMNIB 3 (−7.2 kcal mol^−1^) forms a hydrogen bond with ARG159, π-sigma with VAL179 and π-alkyl with VAL99 (Fig. 11C). Next, the 5E8Q protein from *E. coli*, 3-NBBMNIB 4 (−9.1 kcal mol^−1^) (Fig. 12A) exhibits strong binding affinity by forming a hydrogen bond with TRP22, π–π stacking with PHE31, and π-alkyl interactions with ILE50 and ALA7. BNBMNIB 6 (−8.8 kcal mol^−1^) (Fig. 12D) exhibits hydrogen bonds with MET20, LEU24, and TRP22, while DBMNIH 11 (−8.1 kcal mol^−1^) (Fig. 12B) displays hydrogen bonding with ALA7 and interactions with MET20 and PHE31. DBMNIB 3 (−7.7 kcal mol^−1^) (Fig. 12C) shows a hydrogen bond with LYS58. The *Aureus protein*5ELZ follows, BNBMNIB 6 (−9.3 kcal mol^−1^) (Fig. 13A) forms hydrogen bonds with LYS155 and THR172, and π-anion and Π-cation interactions with GLU202 and ASP239, while 3-NBBMNIB 4 (−8.9 kcal mol^−1^) (Fig. 13B) engages in π–π stacked interactions with PHE256 and HIS228. DBMNIB 3 (−8.6 kcal mol^−1^) (Fig. 13C) interacts with TYR240 and ARG244, and DBMNIH 11 (−7.8 kcal mol^−1^) (Fig. 13D) exhibits π-anion interactions with ASP186. As for the protein 5EOE from *Aeruginosa*, DBMNIH 11 (−8.9 kcal mol^−1^) (Fig. 14A) displays hydrogen bonds with LYS36 and π-cation interactions with ARG57, while 3-NBBMNIB 4 (−8.6 kcal mol^−1^) (Fig. 14B) forms hydrogen bonds with ARG57 and ASN257 and a π-anion interaction with GLU170. BNBMNIB 6 (−8.4 kcal mol^−1^) (Fig. 14C) shows hydrogen bonds with ARG57 and a π-cation interaction with LYS36, whereas DBMNIB 3 (−8.0 kcal mol^−1^) (Fig. 14D) interacts with LYS36 and ASN257. The 5HSG protein for *Pneumonia*, BNBMNIB 6 (−9.0 kcal mol^−1^) (Fig. 15A) forms multiple hydrogen bonds with GLN270 and GLN172, along with π-anion interactions with ASP251 and GLU226. DBMNIB 3 (−8.7 kcal mol^−1^) (Fig. 15B) exhibits hydrogen bonding with GLN270 and π–π stacking with PHE46, while 3-NBBMNIB 4 (−8.5 kcal mol^−1^) (Fig. 15C) shows interactions with GLN270, GLN172, and GLU226. DBMNIH 11 (−8.2 kcal mol^−1^) (Fig. 15D) displays hydrogen bonding with GLN270 and π–π stacking with PHE46. These findings illustrate the strong binding potential of 3-NBBMNIB 4 and BNBMNIB 6 across various pathogenic bacterial proteins. The favorable interactions indicate that they could be effective for antibacterial treatments.

### Molecular dynamics simulation

**Fig. 16:**
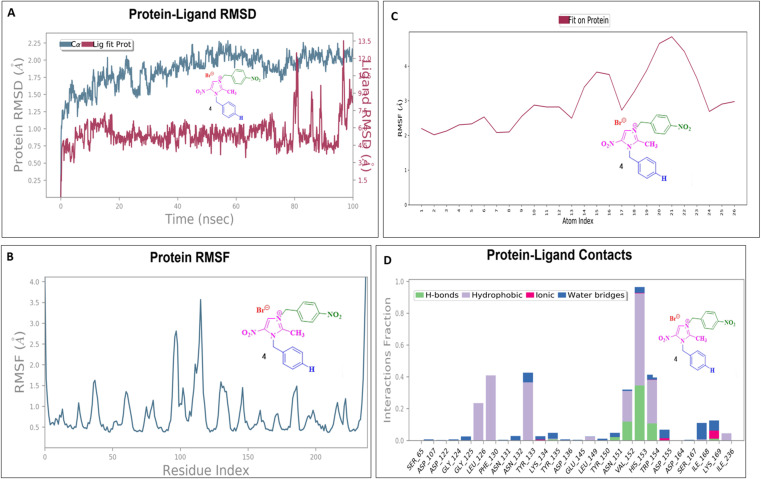
Molecular dynamics simulation of the 5AVA-3-NBBMNIB 4 complex: (A) RMSD analysis of the protein and ligand dynamics; (B) RMSF analysis of the protein residues; highlighting flexibility in the C-terminal region. (C) RMSF of the ligand atoms, indicating the stability and flexibility in atoms 15–21; and (D) protein-ligand contact analysis revealing the key hydrogen, hydrophobic, ionic, and water-mediated interactions.

**Fig. 17:**
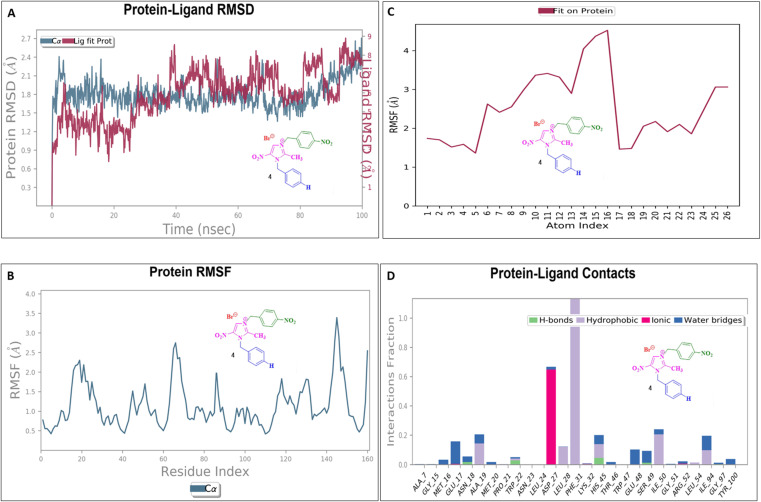
Molecular dynamics simulation of the 5E8Q-3-NBBMNIB 4 complex: (A) RMSD analysis showing the stable protein backbone and ligand flexibility; (B) RMSF analysis of protein residues, with minimal fluctuations and flexible loops; (C) RMSF of ligand atoms, showing moderate fluctuations and flexibility; and (D) protein-ligand contact analysis highlighting the hydrogen bonds, hydrophobic contacts, water bridges, and ionic interactions.

**Fig. 18:**
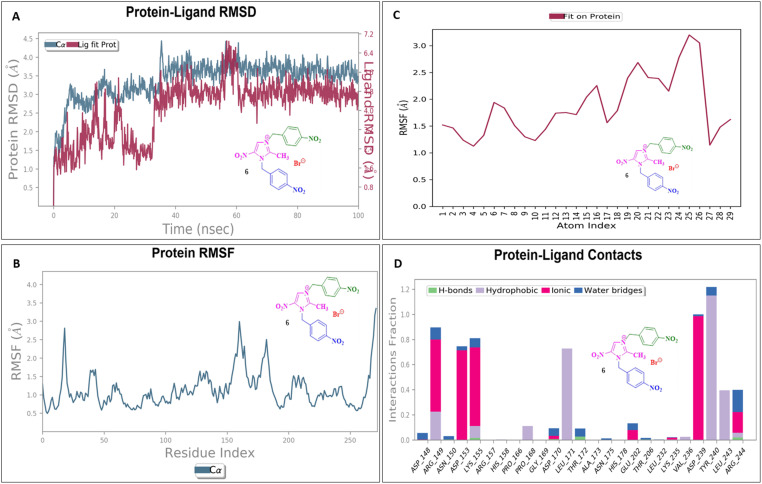
Molecular dynamics simulation of the 5ELZ-BNBMNIB 6 complex: (A) RMSD analysis indicating protein stability and ligand flexibility; (B) RMSF analysis of protein residues showing stability with less deviations; (C) RMSF of ligand atoms indicating moderate stability and flexibility; and (D) protein-ligand contact analysis showing hydrogen bonds, hydrophobic interactions, ionic bonds, and water-mediated contacts.

**Fig. 19:**
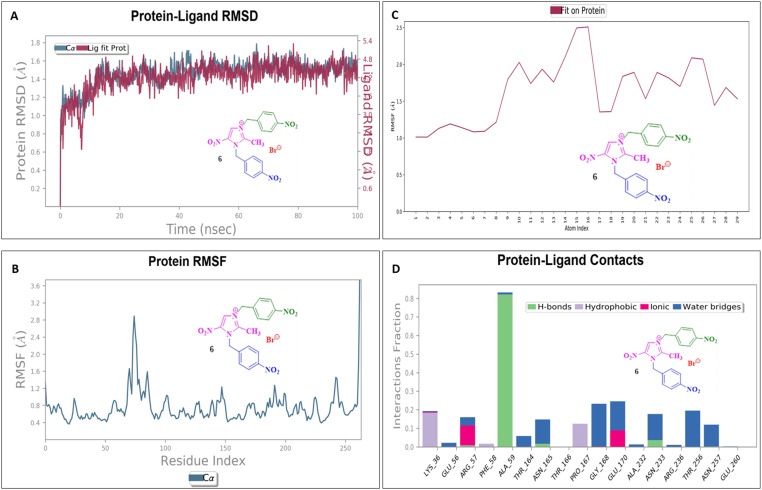
Molecular dynamics simulation of the 5EOE-BNBMNIB 6 complex: (A) RMSD analysis showing stability for both protein and ligand; (B) RMSF analysis of the protein residues, indicating the flexible loop and stable regions; (C) RMSF of the ligand atoms showing stable and flexible regions; and (D) protein-ligand contact analysis revealing hydrogen bonds, hydrophobic contacts, water bridges, and ionic interactions.

**Fig. 20:**
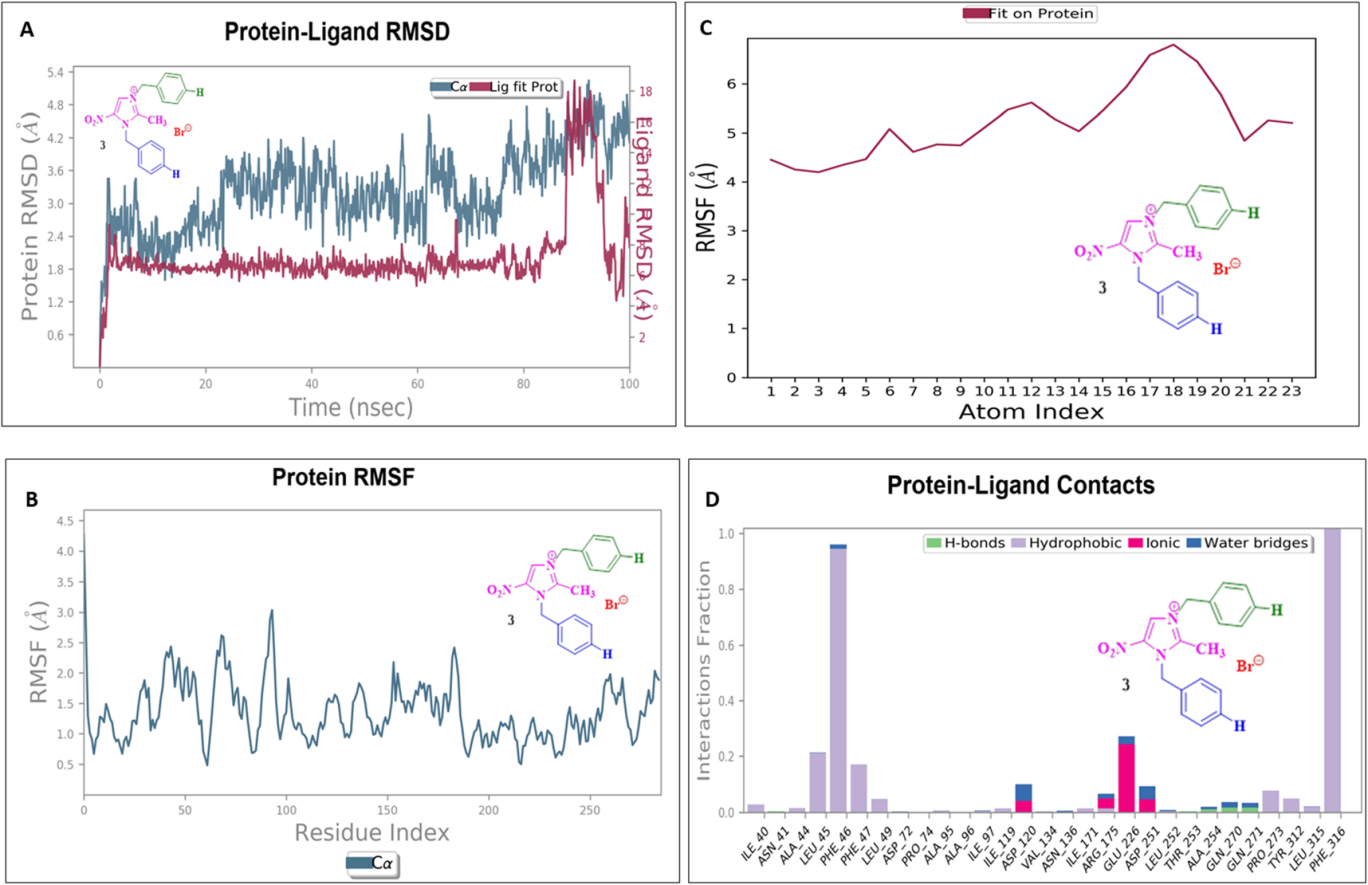
Molecular dynamics simulation of the 5HSG-DBMNIB 3 complex: (A) RMSD analysis showing stability for both protein and ligand, with variations in the ligand dynamics; (B) RMSF analysis of the protein residues; (C) RMSF of ligand atoms indicating flexibility in specific regions; and (D) protein-ligand contact analysis revealing hydrogen bonds, hydrophobic interactions, water bridges, and ionic interactions.

To assess their stability and behavior in a dynamic environment, the top compound for each protein—based on their binding affinities and interactions—was further studied through molecular dynamics simulations. The protein in the 5AVA-3-NBBMNIB 4 complex stays stable (∼1.5–2.0 Å), according to the RMSD analysis (Fig. 16A), while the ligand varies considerably after 80 ns, reaching 13.0 Å, indicating possible separation. While areas close to the C-terminal are more flexible, the protein's RMSF shows stability (Fig. 16B). Atoms 1–10 exhibit ligand RMSF stability (Fig. 16C), while atoms 15–21 exhibit more flexibility. Key hydrogen bonds involving VAL152, HIS153, and TRP154, as well as hydrophobic contacts from LEU126, PHE130, and TYR133 are highlighted by protein-ligand contact analysis as contributing to the complex's stability. Furthermore, the interaction is further stabilized by ionic bonds that form between ASP155 and LYS169. Water-mediated interactions, involving residues like ILE168, LYS169, ASP155, and TYR133, provide additional stabilization (Fig. 16D). Next, the 5E8Q-3-NBBMNIB 4 RMSD analysis (Fig. 17A) reveals a stable protein backbone (Cα) with minimal fluctuations (1.5–2.2 Å) over 100 ns, indicating structural integrity. In contrast, the ligand RMSD progressively increases to 9 Å, suggesting significant flexibility or partial dissociation. Protein RMSF (Fig. 17B) highlights stable residues (RMSF < 2 Å) with higher flexibility around residues 60 and 140, likely corresponding to loops or terminal regions. Ligand RMSF (Fig. 17C) shows moderate fluctuations (1–3 Å) with some regions exceeding 4 Å, indicating flexible functional groups. Key interactions include hydrogen bonds (ASN18, TRP22, HIS45), hydrophobic contacts (ALA19, LEU28, PHE31, ILE50, ILE94), water bridges (MET16, GLY15, HIS45), and ionic bonds (ASP27), supporting stable ligand binding (Fig. 17D). Then, 5ELZ-BNBMNIB 6 RMSD analysis (Fig. 18A) indicates protein stability with fluctuations between 1.0–3.0 Å. Notably, higher deviations (3.5–4.0 Å) are observed during 50–60 ns, followed by stabilization from 60–100 ns. Protein RMSF (Fig. 18B) highlights the overall stability, with minimal fluctuations below 3.0 Å. The ligand RMSD (Fig. 18C) remains within 3.0 Å, showing moderate stability with slight flexibility, attributed to positional alterations on the protein surface. Key interactions include hydrogen bonds with ARG244, hydrophobic contacts with ARG149, LYS155, PRO168, LEU171, ASP239, and TYR240, ionic bonds with ARG149, ASP153, LYS155, and VAL236, and water bridges involving ARG149, LYS155, and ARG244 (Fig. 18D). Thereafter, the 5EOE-BNBMNIB 6 complex demonstrates stable dynamics over a 100 ns simulation. Both components are equilibrated within ∼10 ns. 5EOE maintains an average RMSD of ∼1.5 Å, and BNBMNIB 6 exhibits an average RMSD of ∼1.0 Å, indicating stable protein conformation and binding, respectively (Fig. 19A). A transient increase in the RMSD of compound 6 at around 60 ns suggests a brief conformational change, but the overall complex stability is maintained. The protein RMSF (Fig. 19B) reveals significant flexibility in regions around residues 50, 100, and 250, indicating potential loop or terminal regions. The majority of the protein backbone exhibits lower RMSF values, suggesting a relatively stable core structure. The ligand RMSF (Fig. 19C) analysis shows minimal fluctuations (RMSF ∼1 Å) for residues 1–7, indicating a stable region. A significant increase in flexibility is observed between residues 8 and 16, peaking around residue 15 (RMSF ∼2.5 Å). From residue 17 onwards, the RMSF decreases again, fluctuating between ∼1.3 and ∼2.1 Å, suggesting moderate flexibility in this region. Protein-ligand contact analysis (Fig. 19D) reveals several key interactions. Hydrogen bonds are observed with ALA59 and ASN233. Hydrophobic interactions are formed with LYS36 and PRO167. Water-mediated hydrogen bonds are present with ASN165, GLY168, GLU170, ASN233, THR256, and an unidentified asparagine residue ASN. An ionic interaction is identified between ARG57 and GLU170. Subsequently, dynamics simulations of the 5HSG-DBMNIB 3 complex show that between the first 10 ns, the protein and ligand are in equilibrium. 5HSG shows a stable protein structure with an average RMSD of about 2.0 Å (Fig. 20A). DBMNIB 3 indicates stable binding with an average RMSD of about 1.5 Å. Nonetheless, significant variations in DBMNIB 3 RMSD are noted over the simulation, especially in the 60–80 ns range, suggesting some adaptability in the binding position. The 5HSG-DBMNIB 3 complex stays constant overall in spite of these variations, indicating a dynamic yet enduring relationship. Different regions of flexibility are shown by analyzing the root mean square fluctuation (RMSF) of the protein (Fig. 20B). Three prominent peaks are observed around residues 50, 100, and 250, indicating higher flexibility in these segments, likely corresponding to loop regions or termini. The remaining portions of the protein display lower RMSF values, generally below 1.5 Å, suggesting a more rigid and stable core structure. The RMSF analysis of the ligand (Fig. 20C) reveals varying degrees of flexibility across its atoms. Atoms 1–7 exhibit relatively low fluctuations (RMSF values around 4–4.5 Å), suggesting a more constrained region. A gradual increase in flexibility is observed from atom 8, culminating in a peak around atoms 17–19 (RMSF values reaching approximately 6.5–7 Å), indicating a highly flexible segment. Beyond atom 19, the RMSF values decrease, suggesting a return to more stability towards the ligand. Protein-ligand interaction analysis (Fig. 20D) reveals a network of stabilizing contacts. Hydrogen bonds are observed with ALA254, GLN270, and GLN271. Hydrophobic interactions are formed with ALA44, LEU45, PHE46, PHE47, and LEU49. Water-mediated hydrogen bonds are present with ASP120, ARG175, ASP251, and GLN70. Ionic interactions are identified between ASP120, ARG175, and ASP251.

## Conclusions

In conclusion, this study successfully demonstrated the synthesis of benzylated 2-methyl-5-nitro-imidazolium salts *via N*-alkylation reactions, with the solvent-free silica-supported method significantly reducing the reaction time. Anti-bacterial screening revealed promising inhibition responses, particularly against Gram-negative pathogens. Notably, salts containing the bromide counter anion exhibited excellent anti-bacterial activity. Molecular docking studies provided valuable insights into host–guest interactions between the synthesized salts and various protein sequences. These findings have important implications for the development of novel anti-bacterial agents, and future studies will explore the potential applications of these compounds.

## Experimental sections

### General procedure

AR-grade reagents and reactants were purchased from Merck Chemicals and Sigma-Aldrich Chemicals and used directly without further purification. Proton (^1^H) and carbon-13 (^13^C) NMR spectra were recorded on a Bruker 400 spectrometer (^1^H: 400 MHz, ^13^C: 100 MHz) using CDCl_3_ and DMSO-d_6_ as solvents. The following abbreviations were used to describe multiplicities: s (singlet), d (doublet), t (triplet), q (quartet), and m (multiplet). Chemical shifts are reported in parts per million (ppm) and referenced to tetramethylsilane (TMS). Coupling constants are reported in Hertz (Hz).

### General procedure for *N*-alkylation

2-Methyl-5-nitroimidazole (1.573 × 10^−2^ mmol, 1.0 equiv.) was treated with a slight excess of benzyl bromide/4-nitrobenzyl bromide (1.65 × 10^−2^ mmol, 1.05 equiv.) in the presence of NaOH in CH_3_CN under refluxing conditions for 6–7 h, yielding compounds 1 and 2 in 95–97% yield.

#### 1-Benzyl-2-methyl-5-nitro-1*H*-imidazole 1 (BMNI)

Yield: 8.15 g (95%); mp: 167–169 °C. ^1^H NMR (400 MHz, DMSO-*d*_*6*_): *δ* = 2.6 (s, 3H), 4.52 (s, 2H), 6.9–7.3 (m, 5H), 8.19 (s, 1H). ^13^C NMR (100 MHz, DMSO-*d*_*6*_): *δ* = 12.1, 39.9, 123.4, 127.5, 128.3, 132.7, 139.4, 143.9, 153.7; MS: *m/z*: 217; anal. calcd. for C_11_H_11_N_3_O_2_: calculated: C, 60.82; H, 5.10; N, 19.34. Found: C, 60.78; H, 5.04; N, 19.28.

#### 1-(4-Nitrobenzyl)-2-methyl-5-nitro-1*H*-imidazole 2 (NBMNI)

Yield: 9.89 g (96%); mp: 152–154 °C. ^1^H NMR (400 MHz, DMSO-*d*_*6*_): *δ* = 2.53 (s, 3H), 4.32 (s, 2H), 7.39–7.42 (d, 2H), 8.12–8.16 (d, 2H). ^13^C NMR (100 MHz, DMSO-*d*_*6*_): *δ* = 11.4, 40.5, 124.2, 126.8, 131.4, 142.3, 144.3, 146.7, 153.2; MS: *m/z*: 262; anal. calcd. for C_11_H_10_N_4_O_4_: calculated: C, 50.38; H, 3.84; N, 21.37. Found: C, 50.33; H, 3.80; N, 21.34.

### General procedure for quaternization

*N*-Alkylation reactions were conducted between compounds 1 and 2 (1.0 equiv.) and benzyl bromide/4-nitrobenzyl bromide (1.05 equiv.) in dry CH_3_CN under refluxing conditions for 10–16 h. This resulted in imidazolium bromides 3–6 in 85–88% yield.

### General procedure for solid-supported solvent-free muffle furnace condition

2-Methyl-5-nitroimidazole (1.573 × 10^−2^ mmol, 1.0 equiv.) was treated with a slight excess of benzyl bromide/4-nitrobenzyl bromide (1.65 × 10^−2^ mmol, 1.05 equiv.) using a conventional solvent-free method. The reaction mixture, containing 5 g of silica gel (80–120 mesh), was finely ground using a mortar and pestle. The mixture was then heated in a muffle furnace at 100 °C.

#### 1,3-Dibenzyl-2-methyl-5-nitro-1*H*-imidazolium bromide 3 (DBMNIB)

Yield: 3.37 g (94%); mp: 136–138 °C. ^1^H NMR (400 MHz, DMSO-*d*_*6*_): *δ* = 2.5 (s, 3H), 3.73 (s, 2H), 4.71 (s, 2H), 7.25–7.45 (m, 10H), 8.05 (s, 1H). ^13^C NMR (100 MHz, DMSO-*d*_*6*_): *δ* = 14.5, 42, 53.4, 124.8, 130.5, 132.2, 135.2, 153.3, 154.7, 156.7; MS: *m/z*: 388; anal. calcd. for C_18_H_18_BrN_3_O_2_: calculated: C, 55.68; H, 4.67; N, 10.82. Found: C, 55.64; H, 4.63; N, 10.77.

#### 3-(4-Nitrobenzyl)-1-benzyl-2-methyl-5-nitro-1*H*-imidazolium bromide 4 (3-NBBMNIB)

Yield: 3.96 g (93%); mp: 140–142 °C. ^1^H NMR (400 MHz, DMSO-*d*_*6*_): *δ* = 2.54 (s, 3H), 3.68 (s, 2H), 4.75 (s, 2H), 7.65–7.82 (m, 9H), 8.24 (s, 1H). ^13^C NMR (100 MHz, DMSO-*d*_*6*_): *δ* = 14.2, 41, 54.4, 122.5, 128.2, 130.9, 132.2, 134.9, 137.2, 142.9, 145.3, 149, 152.6, 156.4; MS: *m/z*: 432; anal. calcd. for C_18_H_17_BrN_4_O_4_: calculated: C, 49.90; H, 3.95; N, 12.93. Found: C, 49.86; H, 3.92; N, 12.88.

#### 1-(4-Nitrobenzyl)-3-benzyl-2-methyl-5-nitro-1*H*-imidazolium bromide 5 (1-NBBMNIB)

Yield: 2.93 g (89%); mp: 120–122 °C. ^1^H NMR (400 MHz, DMSO-*d*_*6*_): *δ* = 2.39 (s, 3H), 2.67 (s, 2H), 4.78 (s, 2H), 6.77–7.27 (m, 5H), 7.25–7.27 (d, 2H), 7.92 (s, 1H), 8.22–8.24 (d, 2H). ^13^C NMR (100 MHz, DMSO-*d*_*6*_): *δ* = 13.1, 41.3, 52.7, 116.7, 118.4, 120, 122.4, 124.9, 131.2, 137, 139.4, 146.3, 147.5, 153.3; MS: *m/z*: 432; anal. calcd. for C_18_H_17_BrN_4_O_4_: calculated: C, 49.90; H, 3.95; N, 12.93. Found: C, 49.87; H, 3.90; N, 12.89.

#### 1,3-Bis(4-nitrobenzyl-2-methyl-5-nitro-1*H*-imidazolium bromide 6 (BNBMNIB)

Yield: 3.49 g (96%); mp: 118–120 °C. ^1^H NMR (400 MHz, DMSO-*d*_*6*_): *δ* = 2.32 (s, 3H), 2.86 (s, 2H), 4.77 (s, 2H), 7.39–7.41 (d, 4H), 8.02–8.05 (d, 4H), 8.44 (s, 1H). ^13^C NMR (100 MHz, DMSO-*d*_*6*_): *δ* = 12.8, 42.7, 52.4, 122.8, 130.5, 141.6, 146, 153, 155; MS: *m/z*: 477; anal. calcd. for C_18_H_16_BrN_5_O_6_: calculated: C, 45.20; H, 3.37; N, 14.64. Found: C, 45.16; H, 3.33; N, 14. 61.

### General procedure for anion exchange reaction

*N*-alkylated imidazolium bromides 3–6 (1.0 equiv.) were treated with NaBF_4_, KPF_6_, and LiCF_3_SO_3_ (1.05 equiv.) in 10 mL of deionized water at room temperature with stirring for approximately 1 h, affording the anion-exchanged ionic liquids. Following the anion exchange reaction, the Soxhlet extraction was performed to remove the metal bromide from the ionic liquids using 100 mL of dry THF for approximately 1 hour, yielding ionic liquids 7–18 in 83–89% yield.

#### 1,3-Dibenzyl-2-methyl-5-nitro-1*H*-imidazolium tetrafluoro borate 7 (DBMNIT)

Yield: 245 mg (88%); mp: 115–117 °C. ^1^H NMR (400 MHz, DMSO-*d*_*6*_): *δ* = 2.57 (s, 3H), 3.78 (s, 2H), 4.74 (s, 2H), 7.29–7.51 (m, 10H), 8.09 (s, 1H). ^13^C NMR (100 MHz, DMSO-*d*_*6*_): *δ* = 14.7, 42.3, 53.5, 124.6, 130.8, 132.5, 135.4, 153.6, 154.9, 156.5; MS: *m/z*: 395; anal. calcd. for C_18_H_18_BF_4_N_3_O_2_: calculated: C, 54.71; H, 4.59; N, 10.63. Found: C, 54.67; H, 4.55; N, 10.58.

#### 3-(4-Nitrobenzyl)-1-benzyl-2-methyl-5-nitro-1*H*-imidazolium tetrafluoro borate 8 (3-*NBBMNIT*)

Yield: 233 mg (85%); mp: 129–131 °C. ^1^H NMR (400 MHz, DMSO-*d*_*6*_): *δ* = 2.63 (s, 3H), 3.71 (s, 2H), 4.79 (s, 2H), 7.69–7.86 (m, 9H), 8.28 (s, 1H). ^13^C NMR (100 MHz, DMSO-*d*_*6*_): *δ* = 14.4, 41.7, 54.8, 122.2, 128.7, 130.1, 132.8, 134.5, 137.8, 142.1, 145.6, 149.4, 152.9, 156.1; MS: *m/z*: 440; anal. calcd. for C_18_H_17_BF_4_N_4_O_4_: calculated: C, 49.12; H, 3.89; N, 12.73. Found: C, 49.07; H, 3.85; N, 12.67.

#### 1-(4-Nitrobenzyl)-3-benzyl-2-methyl-5-nitro-1*H*-imidazolium tetrafluoro borate 9 (1-NBBMNIT)

Yield: 231 mg (84%); mp: 125–127 °C. ^1^H NMR (400 MHz, DMSO-*d*_*6*_): *δ* = 2.33 (s, 3H), 2.69 (s, 2H), 4.72 (s, 2H), 6.74–7.23 (m, 5H), 7.23–7.25 (d, 2H), 7.98 (s, 1H), 8.21–8.23 (d, 2H). ^13^C NMR (100 MHz, DMSO-*d*_*6*_): *δ* = 13.7, 41.8, 52.2, 116.1, 118.9, 120.8, 122.7, 124.3, 131.5, 136.7, 139.2, 146.9, 147.6, 153.7; MS: *m/z*: 440; anal. calcd. for C_18_H_17_BF_4_N_4_O_4_: calculated: C, 49.12; H, 3.89; N, 12.73. Found: C, 49.09; H, 3.84; N, 12.87.

#### 1,3-Bis(4-nitrobenzyl-2-methyl-5-nitro-1*H*-imidazolium tetrafluoro borate 10 (BNBMNIT)

Yield: 224 mg (85%); mp: 109–111 °C. ^1^H NMR (400 MHz, DMSO-*d*_*6*_): *δ* = 2.35 (s, 3H), 2.89 (s, 2H), 4.72 (s, 2H), 7.37–7.39 (d, 4H), 8.08–8.11 (d, 4H), 8.41 (s, 1H). ^13^C NMR (100 MHz, DMSO-*d*_*6*_): *δ* = 12.6, 42.3, 52.9, 122.3, 130.7, 141.2, 146.7, 153.4, 155.8; MS: *m/z*: 485; anal. calcd. for C_18_H_16_BF_4_N_5_O_6_: calculated: C, 44.56; H, 3.32; N, 14.44. Found: C, 44.51; H, 3.28; N, 14. 39.

#### 1,3-Dibenzyl-2-methyl-5-nitro-1*H*-imidazolium hexafluoro phosphate 11 (DBMNIH)

Yield: 250 mg (83%); mp: 100–102 °C. ^1^H NMR (400 MHz, DMSO-*d*_*6*_): *δ* = 2.53 (s, 3H), 3.75 (s, 2H), 4.70 (s, 2H), 7.26–7.47 (m, 10H), 8.03 (s, 1H). ^13^C NMR (100 MHz, DMSO-*d*_*6*_): *δ* = 14.8, 42.7, 53.6, 124.4, 130.6, 132.4, 135.7, 153.4, 154.3, 156.9; MS: *m/z*: 453; anal. calcd. for C_18_H_18_F_6_N_3_O_2_P: calculated: C, 47.69; H, 4.0; N, 9.27. Found: C, 47.64; H, 4.03; N, 9.22.

#### 3-(4-Nitrobenzyl)-1-benzyl-2-methyl-5-nitro-1*H*-imidazolium hexafluoro phosphate 12 (3-NBBMNIH)

Yield: 273 mg (88%); mp: 120–122 °C. ^1^H NMR (400 MHz, DMSO-*d*_*6*_): *δ* = 2.58 (s, 3H), 3.63 (s, 2H), 4.76 (s, 2H), 7.62–7.79 (m, 9H), 8.26 (s, 1H). ^13^C NMR (100 MHz, DMSO-*d*_*6*_): *δ* = 14.8, 41.9, 54.3, 122.7, 128.9, 130.4, 132.6, 134.6, 137.4, 142.5, 145.9, 149.5, 152.4, 156.8; MS: *m/z*: 498; anal. calcd. for C_18_H_17_F_6_N_4_O_4_P: calculated: C, 43.38; H, 3.44; N, 11.24. Found: C, 43.34; H, 3.41; N, 11.20.

#### 1-(4-Nitrobenzyl)-3-benzyl-2-methyl-5-nitro-1*H*-imidazolium hexafluoro phosphate 13 (1-NBBMNIH)

Yield: 277 mg (89%); mp: 101–103 °C. ^1^H NMR (400 MHz, DMSO-*d*_*6*_): *δ* = 2.37 (s, 3H), 2.64 (s, 2H), 4.76 (s, 2H), 6.75–7.25 (m, 5H), 7.27–7.29 (d, 2H), 7.94 (s, 1H), 8.27–8.29 (d, 2H). ^13^C NMR (100 MHz, DMSO-*d*_*6*_): *δ* = 13.5, 41.6, 52.4, 116.5, 118.3, 120.7, 122.9, 124.6, 131.8, 137.4, 139.6, 146.1, 147.8, 153.1; MS: *m/z*: 498; anal. calcd. for C_18_H_17_F_6_N_4_O_4_P: calculated: C, 43.38; H, 3.44; N, 11.24. Found: C, 43.34; H, 3.39; N, 11.19.

#### 1,3-Bis(4-nitrobenzyl-2-methyl-5-nitro-1*H*-imidazolium hexafluoro phosphate 14 (BNBMNIH)

Yield: 245 mg (83%); mp: 105–107 °C. ^1^H NMR (400 MHz, DMSO-*d*_*6*_): *δ* = 2.37 (s, 3H), 2.82 (s, 2H), 4.79 (s, 2H), 7.35–7.37 (d, 4H), 8.06–8.09 (d, 4H), 8.47 (s, 1H). ^13^C NMR (100 MHz, DMSO-*d*_*6*_): *δ* = 12.1, 42.5, 52.2, 122.5, 130.3, 141.9, 146.2, 153.9, 155.1; MS: *m/z*: 543; anal. calcd. for C_18_H_16_F_6_N_5_O_6_P: calculated: C, 39.79; H, 2.97; N, 12.89. Found: C, 39.75; H, 2.91; N, 12.86.

#### 1,3-Dibenzyl-2-methyl-5-nitro-1*H*-imidazolium trifluoro methane sulfonate 15 (DBMNIT)

Yield: 265 mg (87%); mp: 97–99 °C. ^1^H NMR (400 MHz, DMSO-*d*_*6*_): *δ* = 2.55 (s, 3H), 3.74 (s, 2H), 4.76 (s, 2H), 7.22–7.43 (m, 10H), 8.07 (s, 1H). ^13^C NMR (100 MHz, DMSO-*d*_*6*_): *δ* = 14.3, 42.5, 53.2, 124.9, 130.4, 132.7, 135.8, 153.6, 154.8, 156.8; MS: *m/z*: 457; anal. calcd. for C_19_H_18_F_3_N_3_O_5_S: calculated: C, 49.89; H, 3.97; N, 9.19. Found: C, 49.84; H, 3.93; N, 9.14.

#### 3-(4-Nitrobenzyl)-1-benzyl-2-methyl-5-nitro-1*H*-imidazolium trifluoro methane sulfonate 16 (3-NBBMNIT)

Yield: 223 mg (89%); mp: 125–127 °C. ^1^H NMR (400 MHz, DMSO-*d*_*6*_): *δ* = 2.52 (s, 3H), 3.65 (s, 2H), 4.71 (s, 2H), 7.67–7.84 (m, 9H), 8.21 (s, 1H). ^13^C NMR (100 MHz, DMSO-*d*_*6*_): *δ* = 14.6, 41.3, 54.6, 122.9, 128.5, 130.5, 132.1, 134.7, 137.5, 142.6, 145.9, 149.7, 152.1, 156.7; MS: *m/z*: 402; anal. calcd. for C_19_H_17_F_3_N_4_O_7_S: calculated: C, 45.42; H, 3.41; N, 11.15. Found: C, 45.38; H, 3.36; N, 11.12.

#### 1-(4-Nitrobenzyl)-3-benzyl-2-methyl-5-nitro-1*H*-imidazolium trifluoro methane sulfonate 17 (1-NBBMNIH)

Yield: 276 mg (88%); mp: 104–106 °C. ^1^H NMR (400 MHz, DMSO-*d*_*6*_): *δ* = 2.35 (s, 3H), 2.62 (s, 2H), 4.75 (s, 2H), 6.79–7.29 (m, 5H), 7.29–7.31 (d, 2H), 7.96 (s, 1H), 8.29–8.71 (d, 2H). ^13^C NMR (100 MHz, DMSO-*d*_*6*_): *δ* = 13.9, 41.4, 52.8, 116.4, 118.7, 120.3, 122.1, 124.5, 131.7, 137.8, 139.8, 146.6, 147.2, 153.9; MS: *m/z*: 502; anal. calcd. for C_19_H_17_F_3_N_4_O_7_S: calculated: C, 45.42; H, 3.41; N, 11.15. Found: C, 45.36; H, 3.39; N, 11.09.

#### 1,3-Bis(4-nitrobenzyl-2-methyl-5-nitro-1*H*-imidazolium trifluoro methane sulfonate 18 (BNBMNIT)

Yield: 298 mg (89%); mp: 99–101 °C. ^1^H NMR (400 MHz, DMSO-*d*_*6*_): *δ* = 2.39 (s, 3H), 2.83 (s, 2H), 4.75 (s, 2H), 7.34–7.37 (d, 4H), 8.05–8.08 (d, 4H), 8.46 (s, 1H). ^13^C NMR (100 MHz, DMSO-*d*_*6*_): *δ* = 12.4, 42.6, 52.5, 122.4, 130.2, 141.3, 146.4, 153.5, 155.9; MS: *m/z*: 547; anal. calcd. for C_19_H_16_F_3_N_5_O_9_S: calculated: C, 41.69; H, 2.95; N, 12.79. Found: C, 41.63; H, 2.91; N, 12.75.

### Binding site prediction in pathogenic bacterial proteins through docking

The protein structures of pathogenic bacteria (PDB IDs: 5AVA, 5E8Q, 5ELZ, 5EOE, and 5HSG) were obtained from the Protein Data Bank (PDB) in PDB format and processed for docking using AutoDock Vina in PyRx 0.8. This preparation included removing water molecules and co-crystallized ligands, adding hydrogen atoms, and assigning Kollman charges to generate the macromolecule prior to docking. Water molecules and co-crystallized ligands were removed, hydrogen atoms were added, and Kollman charges were assigned to generate macromolecules suitable for docking. Open Babel was used to prepare the synthesized compounds 3, 4, 6, and 11, which were supplied in SDF format. For AutoDock Vina compatibility, the structures were translated to PDBQT format with Gasteiger charges and atom types after the compounds' geometries were optimized using energy reduction using the MMFF94 force field. A grid box enclosing the entire protein surface was used for blind docking in order to evaluate possible binding sites. The poses with the highest binding affinity scores were kept to evaluate stability and protein-ligand interactions.

### Assessing structural stability through molecular dynamics simulation

The top-scoring protein-ligand complexes then underwent 100 ns molecular dynamics (MD) simulations using Desmond (D. E. Shaw Research) after virtual screening with AutoDock Vina. The system was set up using Maestro's (Schrödinger, LLC) System Builder tool. Each complex was solvated in an orthorhombic box of TIP3P water molecules with a buffer of 10 Å between the complex and box edges. The system charge was neutralized by adding counter-ions (Na^+^ or Cl^−^). The OPLS_2005 force field was applied to the protein and ligand, and the TIP3P model was utilized for water. Then, the system was subjected to energy minimization, which combines both unconstrained and restrained minimization phases. In the NPT ensemble (300 K, 1 atm), MD simulations were performed with the Nose–Hoover thermostat and the Martyna–Tobias–Klein barostat for temperature and pressure control, respectively. The boundary conditions were implemented on regular intervals. The RESPA integrator was used, with a time step of 2 fs for bound interactions and 6 fs for non-bonded interactions. The Particle Mesh Ewald (PME) approach was used to treat long-range electrostatics, with a 9 Å cutoff for van der Waals and short-range electrostatic interactions. Simulation Interaction Diagrams (SIDs) and RMSD and RMSF calculations were used to examine protein-ligand interactions in order to evaluate the residue flexibility and complex stability.

## Conflicts of interest

There are no conflicts to declare.

## Supplementary Material

RA-015-D5RA04144A-s001

## Data Availability

The data supporting this article have been included within this manuscript and its SI. Supplementary information is available. See DOI: https://doi.org/10.1039/d5ra04144a.
