# Supplementary material for: Exploring the anti-microbial potential of benzylated imidazolium salts: synthesis, docking studies, and biological evaluation
Source: RSC Adv. 2025 Sep 24;15(42):35115–36. doi: 10.1039/d5ra04144a (PMC12459267; doi:10.1039/d5ra04144a)

Supplementary Information (SI) for RSC Advances.
This journal is © The Royal Society of Chemistry 2025

Exploring the Anti-Microbial Potential of Benzylated Imidazolium Salts: Synthesis, Docking Studies, and Biological Evaluation

Pandurangan Ganapathi [a], Kilivelu Ganesan [a*], Nallusamy Vijaykanth[b], Srisailas Muthialu[c], Shiek SSJ Ahmed[d], Mohammed Mujahid Alam[e], Mohamed Hussien[e]

[a] PG & Research Department of Chemistry, Presidency College (Autonomous), Chennai 600 005, India.
[b] Filaria / Malaria Clinic, Government Hospital, Valangaiman, Thiruvarur District-612804, Tamil Nadu, India.
[c] Vasi Pharma LLC, 150 N Research Campus Drive, Kannapolis, NC 28081, USA
[d] Drug Discovery and Multi-omics Laboratory, Faculty of Allied Health Sciences, Chettinad Hospital and Research Institute, Chettinad Academy of Research and Education, Kelambakkam - 603103, Tamil Nadu, India.
[e] Department of Chemistry, College of Science, King Khalid University, P. O. Box 9004, Abha, 61413, Saudi Arabia.
Email: kiliveluganesan@yahoo.co.in

^{1}H NMR spectrum of 1,3-dibenzyl-2-methyl-5-nitro-1*H*-imidazoliumbromide **3**

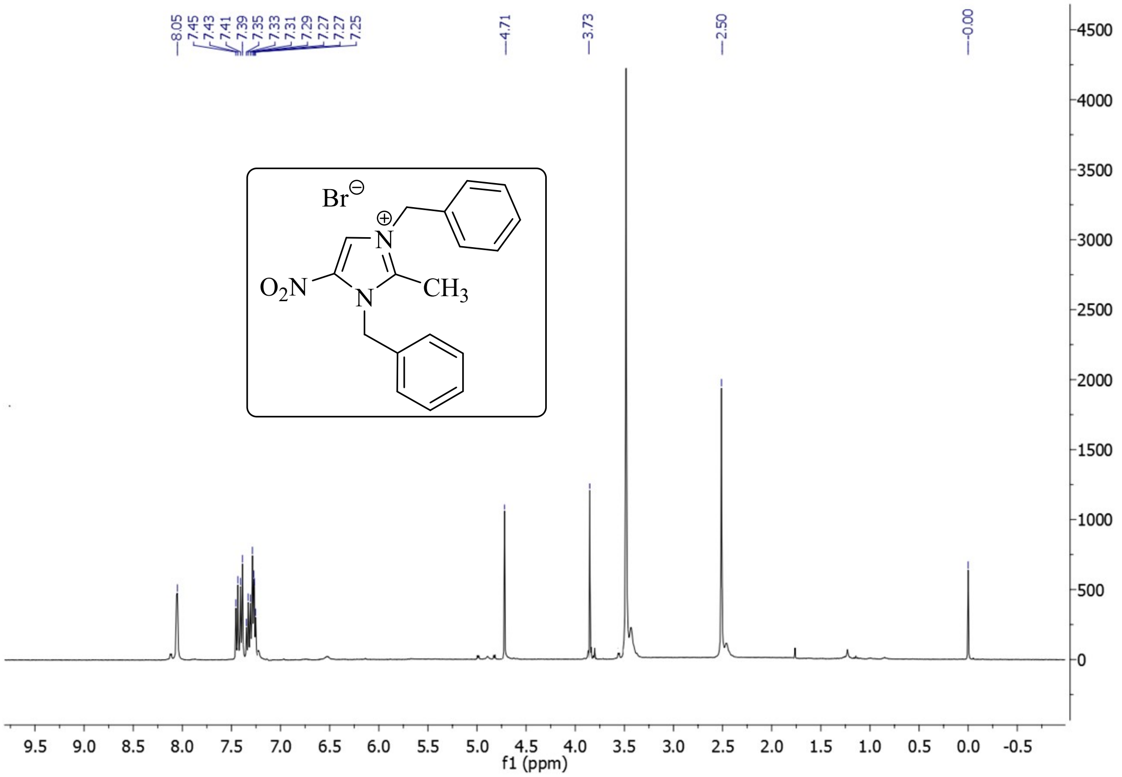

^{13}C NMR spectrum of 1,3-dibenzyl-2-methyl-5-nitro-1*H*-imidazoliumbromide **3**

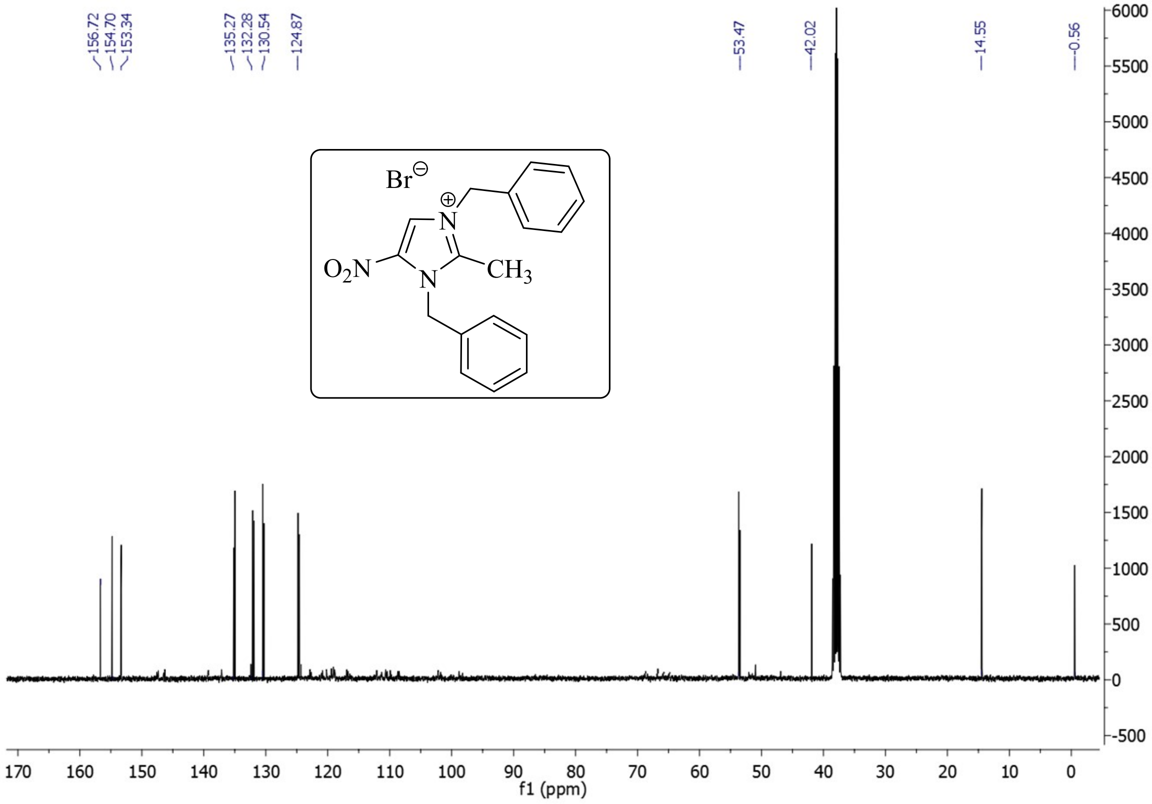

^{1}H NMR spectrum of 3-(4-nitrobenzyl)-1-benzyl-2-methyl-5-nitro-1*H*-imidazoliumbromide **4**

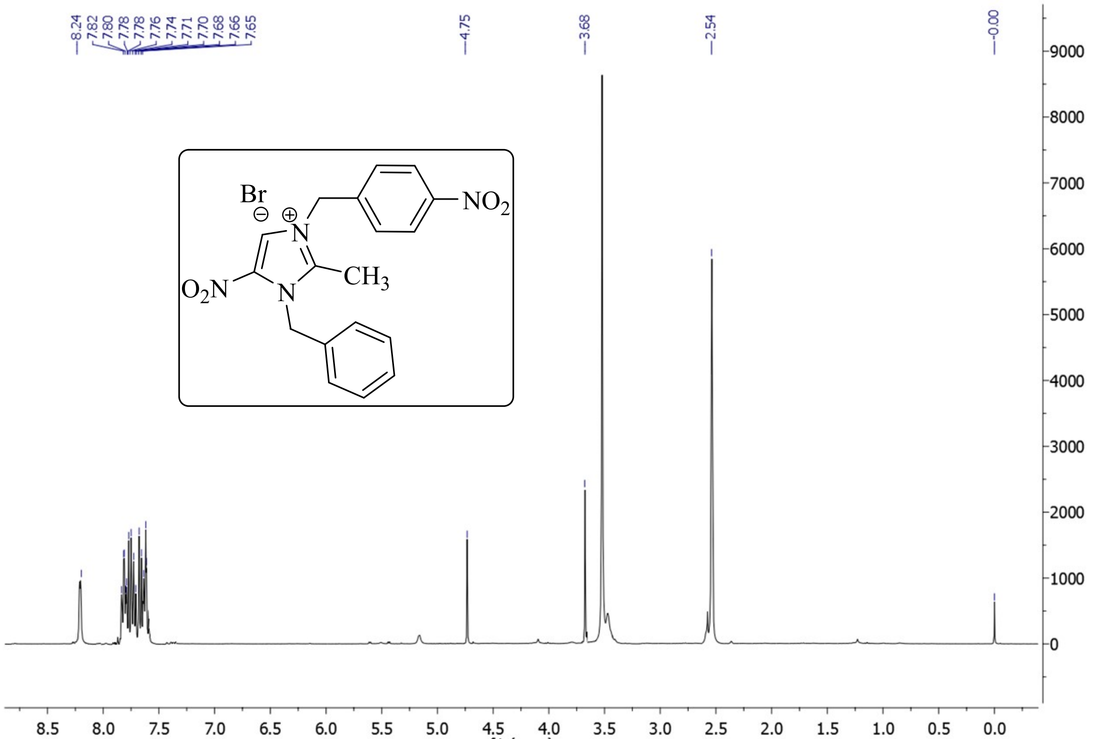

^{13}C NMR spectrum of 3-(4-nitrobenzyl)-1-benzyl-2-methyl-5-nitro-1*H*-imidazoliumbromide **4**

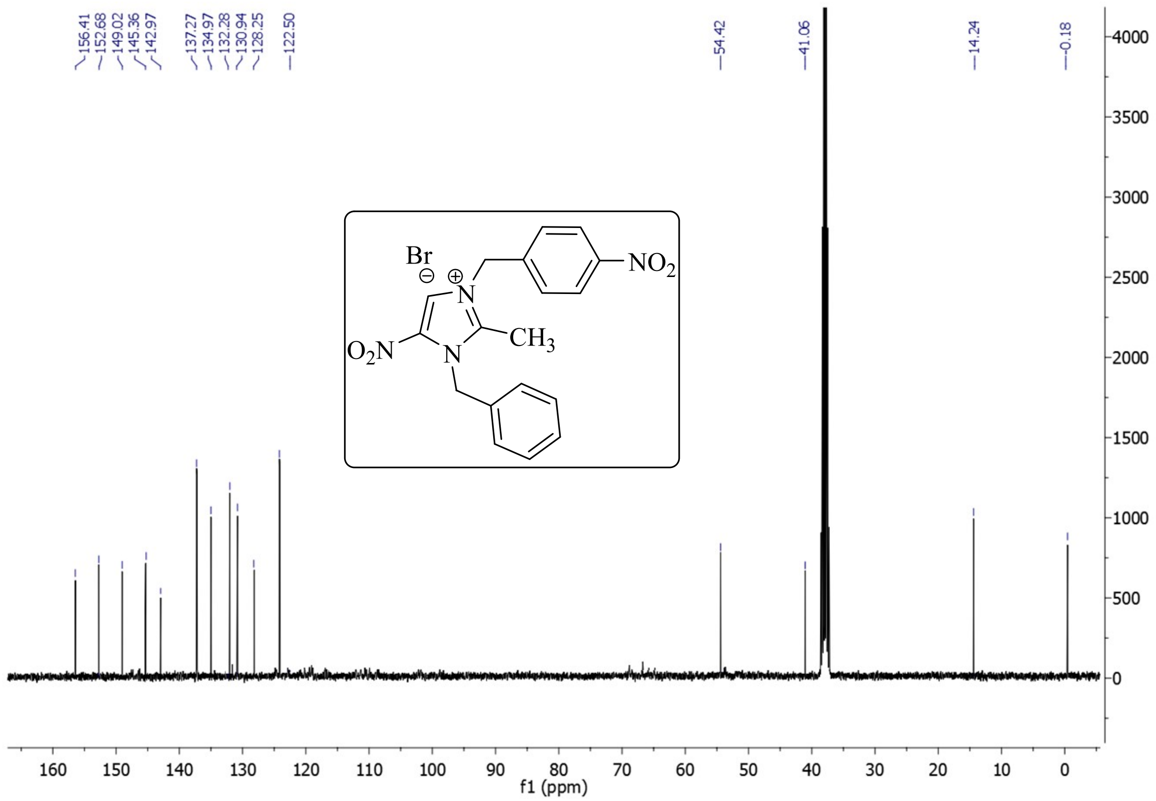

^{1}H NMR spectrum of 1-(4-nitrobenzyl)-3-benzyl-2-methyl-5-nitro-1*H*-imidazoliumbromide **5**

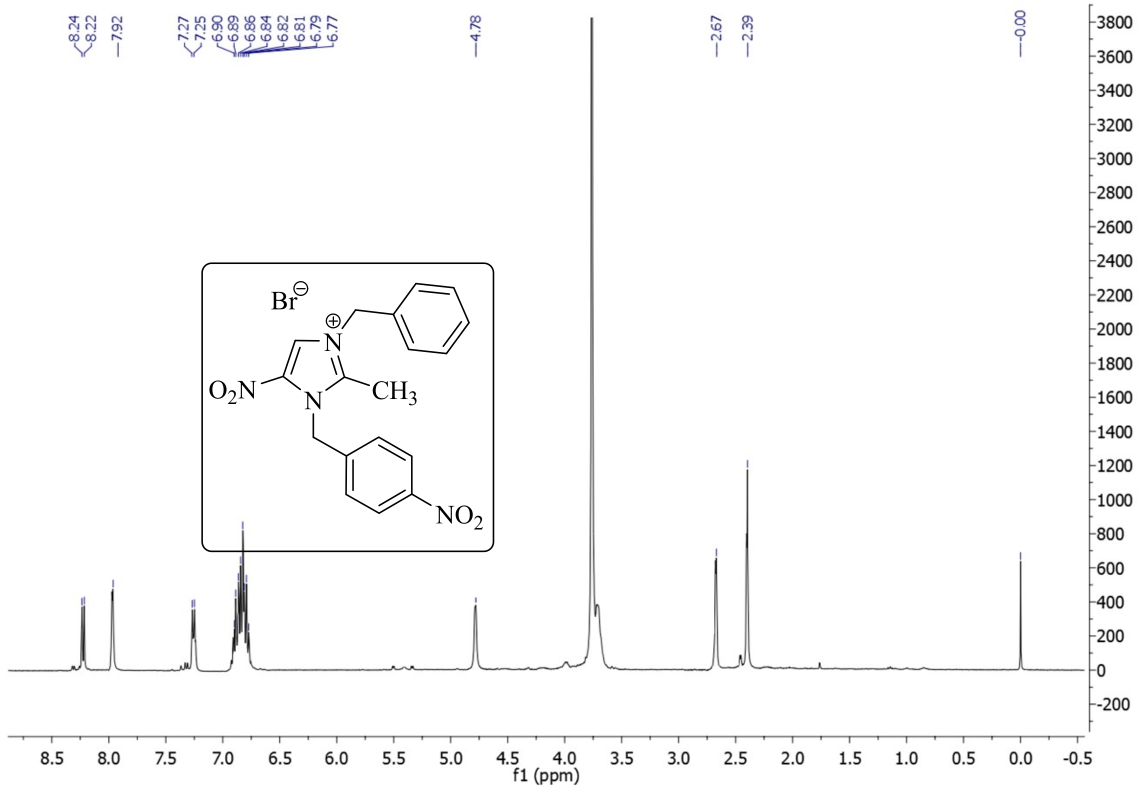

^{13}C NMR spectrum of 1-(4-nitrobenzyl)-3-benzyl-2-methyl-5-nitro-1*H*-imidazoliumbromide **5**

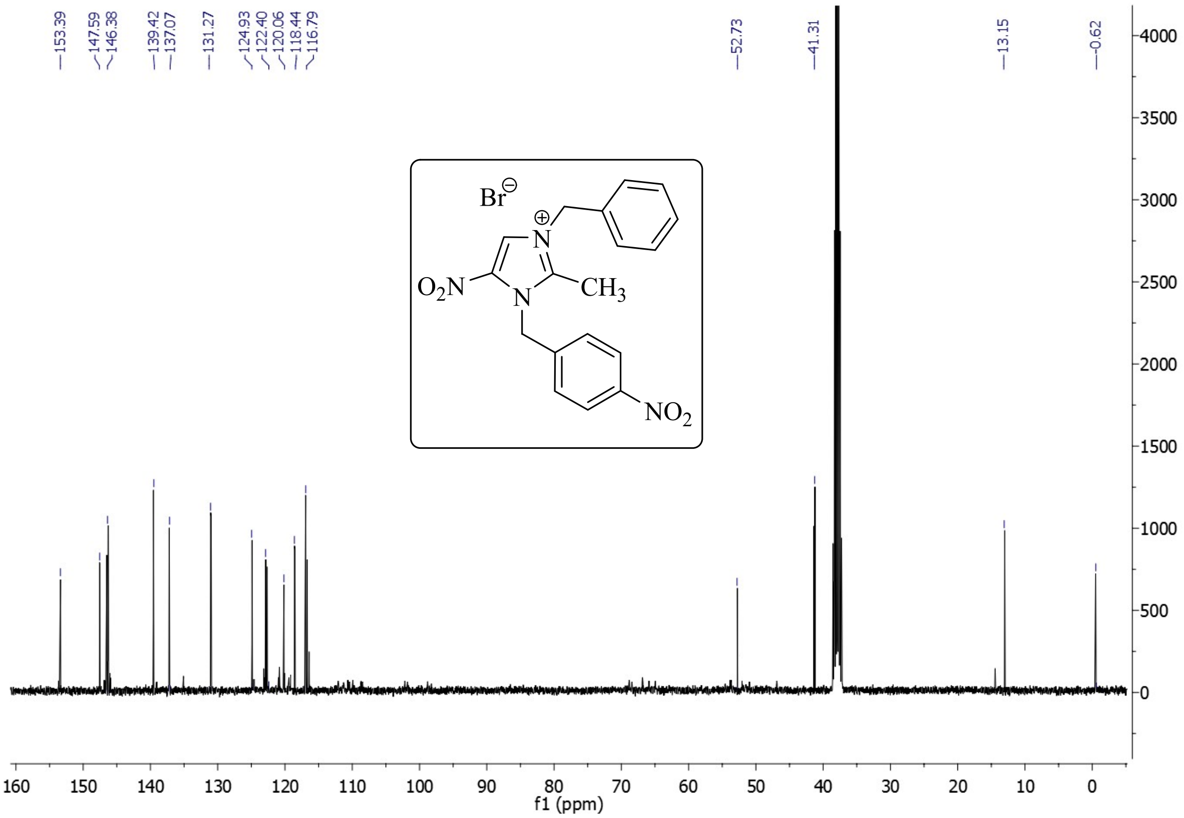

^1H NMR spectrum of 1,3-bis(4-nitrobenzyl-2-methyl-5-nitro-1*H*-imidazoliumbromide **6**

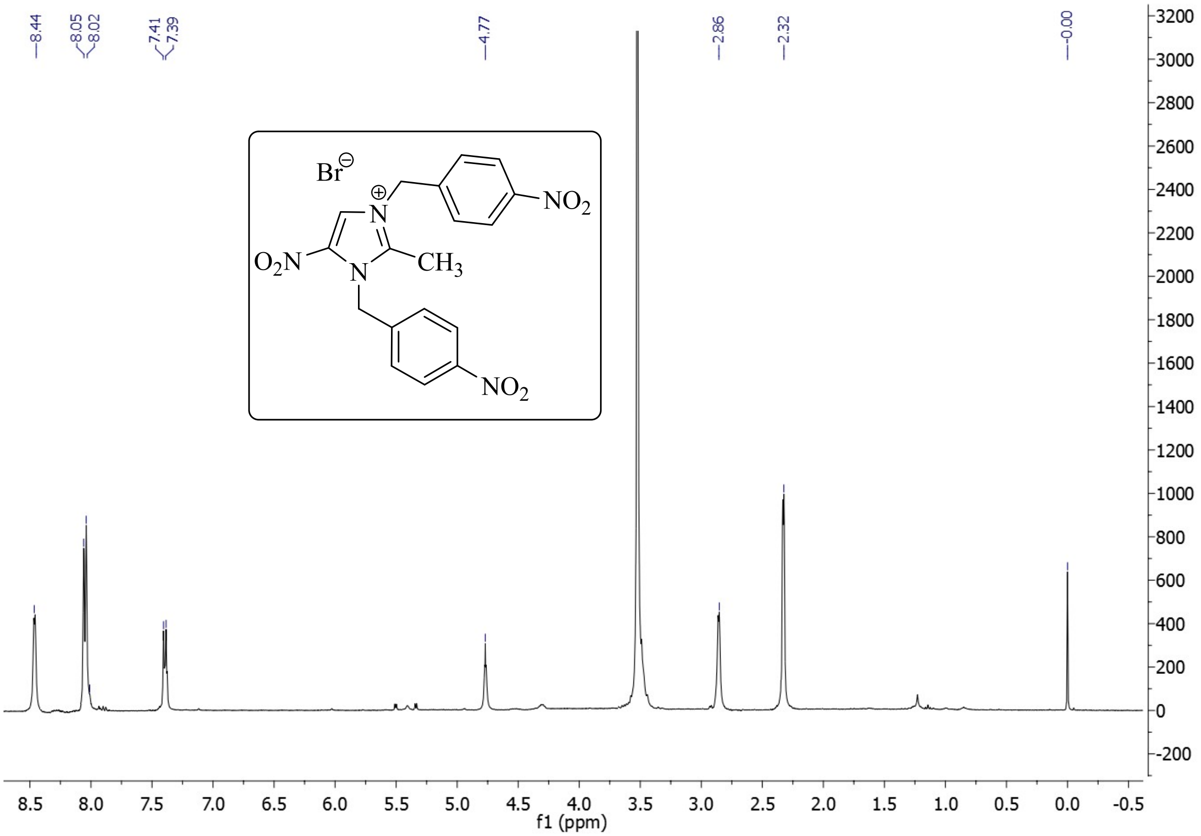

^{13}C NMR spectrum of 1,3-bis(4-nitrobenzyl-2-methyl-5-nitro-1*H*-imidazoliumbromide **6**

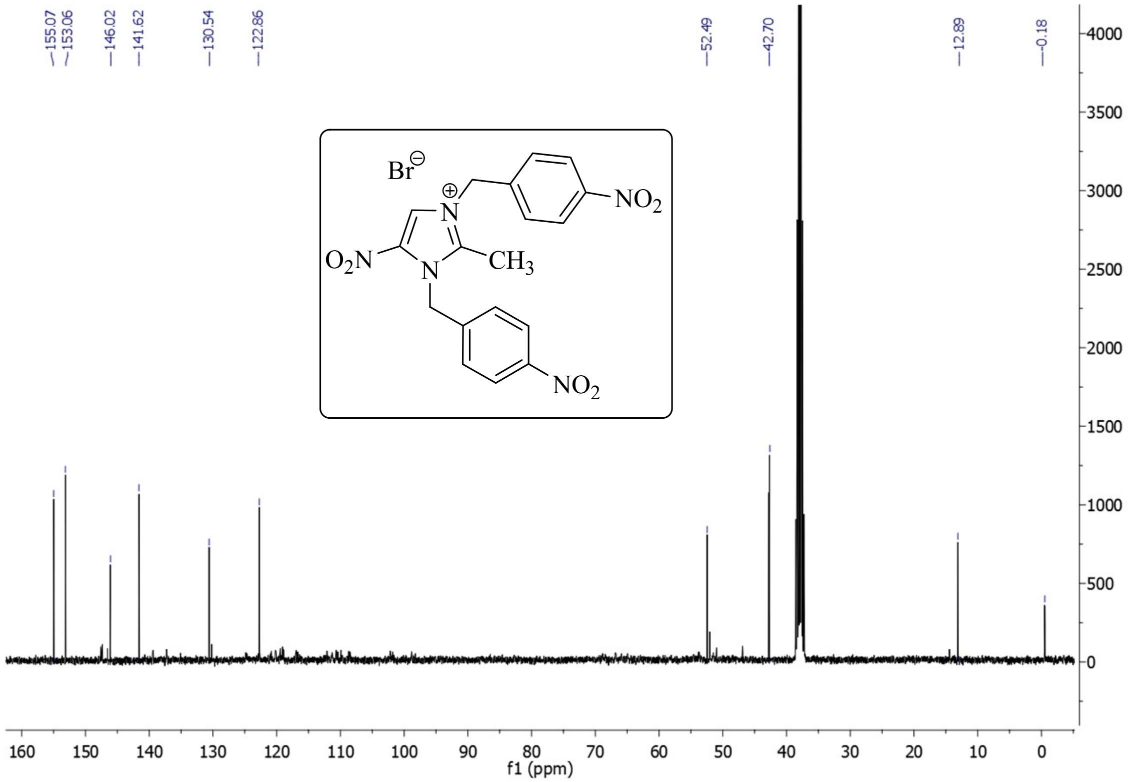

Supplement: RA-015-D5RA04144A-s001 [file RA-015-D5RA04144A-s001.pdf]
